# Screening and Verification of Differentially Expressed Long Non-coding RNAs in the Peripheral Blood of Patients With asthma

**DOI:** 10.3389/fphar.2022.834009

**Published:** 2022-02-22

**Authors:** Cheng Ma, Shiyuan Wang, Yuxue Cao, Weifeng Tang, Tulake Wuniqiemu, Fangzhou Teng, Xueyi Zhu, Ying Wei, Jingcheng Dong

**Affiliations:** ^1^ Department of Integrative Medicine, Huashan Hospital, Fudan University, Shanghai, China; ^2^ Institutes of Integrative Medicine, Fudan University, Shanghai, China

**Keywords:** asthma, full transcriptome sequencing, bioinformatics, lncRNAs, mRNAs

## Abstract

Growing evidence suggests that long non-coding RNAs (lncRNAs) play a key role in the pathogenesis of asthma. Although some differentially expressed lncRNAs have been identified in asthmatic patients, many asthma-related lncRNAs have not been annotated. In the present study, six patients and three healthy subjects were randomly selected from 34 asthmatic patients and 17 healthy subjects. Second-generation high-throughput sequencing was performed on their peripheral blood samples. There were 1,137 differentially expressed lncRNAs in the asthma patients compared to in the healthy controls, of which 485 were upregulated and 652 were downregulated. The top 30 enriched GO and KEGG terms were identified, and the cytosolic ribosome (GO:0022626) and ribosome (hsa03010) were associated with the most differentially expressed lncRNAs. The top 10 differentially expressed lncRNAs associated with asthma were verified by an lncRNA-mRNA co-expression network and RT-qPCR. Seven of the these (NONHSAT015495.2, MSTRG.71212.2, NONHSAT163272.1, NONHSAT181891.1, NONHSAT190964.1, ENST00000564809, and NONHSAT076890.2) were down-regulated in the peripheral blood of asthmatic patients, which was consistent with the sequencing results. Three patients and three healthy subjects were randomly selected from the remaining subjects to verify these seven lncRNAs by RT-qPCR, which further confirmed the sequencing results. Public database GSE106230 was also in agreement with the FPKM (Fragments Per kilobase of exon model per Million mapped reads) trends of ENST00000564809, NONHSAT015495.2, NONHSAT181891.1, and NONHSAT190964.1. In conclusion, the present study identified seven lncRNAs that may serve as potential biological markers for asthma.

## Introduction

Asthma is a heterogeneous disease characterized by chronic airway inflammation, airway hyperresponsiveness, and airway remodeling (Hammad and Lambrecht, 2021), and affects more than 300 million patients worldwide (Côté et al., 2020). The pathogenesis of asthma remains unclear. It is generally thought that asthma is caused by a combination of genetic and environmental factors, resulting in an imbalance in the ratio of T helper cells (Th)1/Th2 and Th17/Treg cytokines and other factors in the airway, resulting in persistent inflammation and hyperresponsiveness (Kudo et al., 2012; Fahy, 2015).

Long non-coding RNAs (lncRNAs) are a type of non-coding RNA that are >200 nucleotides in length, and can interact with proteins, DNA, or RNA (Chen and Shan, 2020; Vydzhak et al., 2020). LncRNAs are involved in the regulation of many biological processes, including chromatin modification, transcriptional activation and inhibition, post-transcriptional modification, and nuclear transport, and as a result play a role in many important biological processes, including cell differentiation and development (Lodde et al., 2020). Recent studies have shown that lncRNAs play a key role in the pathogenesis of asthma. For instance, the overexpression of lncRNA-MALAT1 is associated with asthma patients, and its expression was negatively correlated with the expression of Th1/Th2 (Liang and Tang, 2020). Another lncRNA, MEG3, is a competing endogenous RNA that regulates the balance of Treg/Th17 in asthma patients (Qiu et al., 2019).

With the recent development and continuous improvement of second-generation high-throughput sequencing technology, current transcriptomics research depends on accurate and rapid screening for relevant lncRNAs that are related to specific diseases. An increasing number of lncRNAs have been functionally annotated. Although studies have reported some differences in lncRNAs in asthma patients during both childhood (Zheng et al., 2020) and adulthood (Gál et al., 2020), there are many lncRNAs with an unclear relationship to asthma that require investigation and annotation. In the present study, we aimed to perform whole transcriptome sequencing in patients with asthma using second-generation high-throughput sequencing to identify dysregulated lncRNAs that are associated with asthma. We will then perform GO and KEGG enrichment analysis to identify potential biological functions associated with the dysregulated lncRNAs. In doing so, we hope to identify an lncRNA signature for asthma that may have clinical diagnostic or prognostic applications, and may alleviate the burden on healthcare systems by enabling timeous and effective interventions.

## Methods

### Subjects

#### Study Cohort

Subjects were patients with asthma who attended the Outpatient of Integrative Medicine, Huashan Hospital, Fudan University (from 1 November 2019 to 31 January 2020), and healthy volunteers who were recruited through clinical research recruitment advertisements. According to the inclusion and exclusion criteria (detailed below), a total of 34 subjects in the asthma group and 17 subjects in the healthy control group were included. Each subject signed an informed consent form.

#### Diagnostic Criteria for Asthma

The diagnostic criteria for asthma were based on the 2016 Chinese Medical Association Respiratory Diseases Association Bronchial Asthma Prevention and Control Guidelines. 1) Clinical symptoms and signs of typical asthma: recurrent wheezing, shortness of breath, with or without chest tightness or coughing, especially at night and in the morning, and often attacks after exposure to allergens such as cold air, physical or chemical irritation, upper respiratory tract infection, or exercise. Sporadic or diffuse wheezing can be heard in both lungs during attack, with a predominant expiratory phase and prolonged expiratory phase. The above symptoms or signs are relieved spontaneously or after treatment. 2) Objective inspection of variable airflow limitation: 1) a positive bronchial diastolic test with an increase in FEV between 1% > 12% after bronchodilator inhalation, and an increase in absolute FEV between 1 > 200 ml 2) A positive bronchial provocation test. 3) Peak expiratory flow (PEF) average daily diurnal variation rate of >10% or weekly PEF variation rate of >20%. 4) The patient meets the “typical clinical symptoms and signs of asthma,” and has any of the “objective examinations of variable airflow limitation,” and wheezing, shortness of breath, chest tightness, or cough caused by other diseases are excluded.

### Inclusion and Exclusion Criteria

#### Selection Criteria for Asthma Patients

(1) Diagnosed as bronchial asthma according to 2016 Respiratory Diseases Association Bronchial Asthma Prevention and Control of Chinese Medical Association and Guidelines and 2019 Global Initiative for Asthma (GINA) guidelines (2) The selected age range was 30–55 years, regardless of gender. (3) No history of respiratory diseases, except for asthma. (4) No upper/lower respiratory tract infections and no antibiotic treatment within 4 weeks; no oral/intravenous glucocorticoid treatment or other respiratory medication except Inhaled Corticosteroids/Long-Acting Beta2 Agonist (ICS/LABA) and Short-Acting Beta2-Agonists (SABA) within 4 weeks. (5) No smoking history or had quit smoking for more than 12 months. (6) Voluntarily participated in the study and signed informed consent forms.

### Selection Criteria for Healthy Subjects

(1) The selected age range was 30–55 years, regardless of gender. (2) The physical examination results were normal, without any abnormal symptoms or signs. (3) Laboratory/imaging examination results were normal or abnormal, but had no clinically meaningful results. (4) No smoking history or had quit smoking for more than 12 months. (5) Voluntarily participated in the study and signed informed consent forms.

### Exclusion Criteria

#### Exclusion Criteria for Asthma Patients

(1) Patients with severe persistent and acute exacerbation of asthma requiring oral or intravenous corticosteroid treatment. (2) Patients with COPD, bronchiectasis, interstitial lung disease, active *tuberculosis*, pulmonary embolism, pleural effusion, or other lung diseases. (3) Patients with cardiovascular, cerebrovascular, digestive, endocrine, kidney, urinary, blood, and other systemic diseases or malignant tumors. (4) Patients who received antibiotic treatment within 4 weeks of the study. (5) Patients requiring treatment with glucocorticoids or immunosuppressive agents. (6) Patients who have been pregnant or breast fed within 12 months (8) Patients participating in other clinical studies during the same period. (9) Patients who refused to sign informed consent forms.

#### Exclusion Criteria for Healthy Subjects

(1) Subjects who received antibiotics or glucocorticoid therapy within 4 weeks of the study. (2) Subjects who had been pregnant or had breast fed within 12 months. (3) Subjects participating in other clinical studies during the same period. (4) Subjects who refused to sign informed consent forms.

### Ethical Review

This single-center, prospective, and exploratory clinical study was approved by the Ethical Review Committee of Huashan Hospital Affiliated to Fudan University. Ethical approval number (2019). Temporary Approval No (593). The study was registered with the China Clinical Trial Registration Center, clinical research registration number: ChiCTR2000031267. The study was performed according to the guidelines established by the Declaration of Helsinki.

### Research Methods

#### Clinical Characteristics

Detailed clinical consultations were conducted on asthma patients and healthy subjects, with a comprehensive physical examination performed, including body temperature, pulse, respiration, blood pressure, weight, and height. Medical histories, including allergies and other medical conditions, were obtained to confirm the asthma diagnosis. Routine blood, urine, liver and kidney function, blood sugar, peripheral blood EOS count, blood total IgE, lung function, and FeNO tests were performed for all subjects.

#### Determination of Lung Function and FeNO

Both lung function and FeNO tests were performed between 8:00–11:00 a.m. Subjects were required to stop vigorous activities for 2 h before the test and stay at rest for 15 min. Patients with asthma were tested before the administration of asthma control drugs. Both lung function and FeNO measurement were tested using the Pulmonary Function Room at Huashan Hospital Affiliated to Fudan University.

#### Detection of Inflammatory Cytokines

Venous blood (4 ml) was collected from fasting subjects before 8:00 a.m., and centrifuged at 4,000 rpm for 10 min at room temperature to separate red blood cells and serum. The serum was removed and stored in 2 ml Eppendorf tubes at −80°C. An ELISA was used to detect serum inflammatory factors (IL-4, IL-5, IL-8, IL-9, IL-13, IL-17A, IL-25, IL-33, and IFN-γ) and eotaxin.

#### Total RNA Extraction, Library Construction and Whole Transcriptome Sequencing

Venous blood (2.5 ml) was collected from fasting subjects before 8:00 a.m. Each sample was collected in a disposable vacuum blood RNA collection tube, mixed well, and stored at −80°C. An RNEasy micro kit and RNase-Free DNAse kit were used for the extraction and purification of total RNA, according to the manufacturer’s instructions. A NanoDrop ND-2000 spectrophotometer and Agilent Biochemical Analyzer 2,100 were used to determine RNA quality by measuring RNA integrity number (RIN). The RIN values ranged from to 0 to 10, with higher scores indicating better RNA integrity. RNA samples that passed the quality assessment were used as the templates for first and second strand cDNA synthesis. A Qubit^®^ 2.0 Fluorometer (Invitrogen, Q32866) was used to detect DNA concentration. A 2100 Bioanalyzer (Agilent Technologies) was used to detect the band distribution, and then cluster generation and first-direction sequencing primer hybridization were performed on the cBot for the Illumina sequencing platform, according to the manufacturer’s instructions. The Illumina sequencing reagents were prepared according to manufacturer’s instructions, and a patterned flow cell was used for improved cluster generation. Paired-end sequencing was performed using Illumina software, with real-time data analysis.

#### Bioinformatics Analysis

Seqtk was used to filter the original data to obtain clean reads for data analysis. The spliced mapping algorithm HISAT2 (version 2.0.4) was used for genome mapping of preprocessed sequences. The human GRCh37 assembly (Ensembl) was used for genome mapping. StringTie software (version 1.3.0) was used to count the number of fragments corresponding to each gene after HISAT2 comparison, and then normalized to the trimmed mean M values (TMM) (fragments per kilobase of exon model per million mapped reads, FKPM) for each gene.

The edgeR R package was used to comparatively analyze the differentially expressed genes between the sample groups. After obtaining the *p*-value, multiple testing correction was applied, and the *p*-value threshold was determined by controlling the false discovery rate (FDR), and expressed as a q-value. The fold change (FC) was calculated based on the FPKM value. Cutoffs of q-value ≤ 0.05, and FC ≥ 2 were applied to filter for differentially expressed genes. The differentially expressed genes (DEGs) were mapped to the GO database, and the number of genes corresponding to each entry was calculated. KEGG pathway enrichment analysis was performed similarly for the DEGs. Differentially expressed lncRNAs were sorted in descending |log2FC| value, and the top 50 dysregulated lncRNAs were identified. KEGG pathways related to asthma with a large number of differentially expressed genes were selected, and the mRNAs enriched in these pathways were selected to generate an lncRNA-mRNA co-expression network and identify biologically relevant lncRNAs.

#### Real-Time Quantitative PCR

PCR primers were designed using Primer Express 3.0.1 software, synthesized by Sangon Biotech Co., Ltd (Shanghai, China) and verified by Primer-BLAST. The 2^−ΔΔCT^ method was used to analyze the relative changes in gene expression, with GAPDH serving as the internal reference. Primer sequences and related information were included in Table 1.

**TABLE 1:** RT-qPCR Primer sequence.

No.	Name	Sequence (5′ to 3′)
1	NONHSAT015495.2-F	GCA​CTC​AGG​AGG​CAT​AGC​AAA
	NONHSAT015495.2-R	CGT​GAT​CTC​CCT​GAG​CTC​ATC
2	MSTRG.71212.2-F	GCC​TCA​GCT​CAC​CAT​GTT​GA
	MSTRG.71212.2-R	AGG​GCT​GGC​GAT​CTA​GGA​A
3	NONHSAT163272.1-F	CCA​GTG​ATA​GGC​CTT​GGT​AGC​T
	NONHSAT163272.1-R	TCT​ACA​TGC​ATG​CAG​TCA​TTT​CC
4	ENST00000564809-F	GCC​AAA​GCC​AAC​AGC​ATC​TT
	ENST00000564809-R	GAA​TTG​ATA​ATG​GCG​TGA​AAC​G
5	NONHSAT181891.1-F	TGA​CTG​CAG​TGA​TGA​AGA​AAG​GA
	NONHSAT181891.1-R	CTG​GAG​GTC​CCT​GCT​GAA​TC
6	ENST00000615535-F	TGCCCGGAGCCTGAGA
	ENST00000615535-R	TCCCCTGGCCCCATTT
7	NONHSAT190964.1-F	AGC​ATG​CCA​CAC​ATT​GAT​GAA
	NONHSAT190964.1-R	GTA​ATC​CCG​CCT​TGC​TTT​GA
8	NONHSAT076890.2-F	GCA​GTA​ACC​TTC​ATC​TCT​CTT​CTA​TAG​GT
	NONHSAT076890.2-R	TTC​AAA​GCT​GTC​ACT​CTC​CAT​GTT
9	*GAPDH*-F	CAC​CCT​GTT​GCT​GTA​GCC​AAA
	*GAPDH*-R	CAC​CCT​GTT​GCT​GTA​GCC​AAA

### Statistical Analysis

Statistical analysis was performed using SPSS 25.0 (IBM), and plots were generated using GraphPad 8.0 (GraphPad software, Inc). A *t*-test was used to analyze continuous variables that conformed to a normal distribution, and data are presented as the mean ± standard deviation. Data that did not conform to a normal distribution were log2 transformed, and analyzed by *t*-test. If the log2 transformed data did not conform to a normal distribution, a Wilcoxon test was used, and the median and interquartile intervals were used for statistical analysis. A Chi-squared test was used to compare categorical variables. Differences were considered to be statistically significant at *p* < 0.05.

## Results

### Cohort Clinical Characteristics

The clinical characteristics of the asthma group (n = 34) and the healthy control group (n = 17), such as age, sex, and BMI, were compared to exclude any potential sources of bias from the subsequent analysis. There was a difference in gender composition between the asthma group and the healthy control group (*p* < 0.001), with more female patients (85.29%) than male patients (14.71%) in the asthma group. There were no statistically significant differences between the two groups in terms of age and BMI (*p* > 0.05) (Table 2).

**TABLE 2:** Cohort clinical characteristics.

		Asthma group	Control group	*p* Value
Gender	Male	5 (14.71%)	11 (16.71%)	<0.001
	Female	29 (85.29%)	6 (35.29%)	
Age		42.62 ± 8.16	40.76 ± 6.01	0.411
BMI (kg/m^2^)		24.78 ± 5.31	23.79 ± 3.29	0.422

### Comparison of Lung Function, FeNO and Inflammatory Cytokines

We compared the lung function, FeNO, and inflammatory cytokine levels in the two groups, and found that the lung function indices (FEV1%, FEV1/FVC%, and PEF%) and small airway function indexes (MEF25%, MEF50%, MEF75%, and MMEF75/25%) of the healthy subjects were significantly better than those in the asthma group (*p* < 0.05). Compared with healthy subjects, asthma patients had higher FeNO (*p* < 0.05), total blood IgE (*p* < 0.05), peripheral blood EOS counts (*p* < 0.05), and serum IL-8 levels (*p* < 0.01). The levels of inflammatory cytokines IL-17A, IL-25, IL-33, and eotaxin were higher in the asthma group than in the healthy control group, but these differences were not significant, and there were no significant differences in the levels of IL-4, IL-5, IL-9, and IL-13 between the two groups (Table 3).

**TABLE 3:** Comparison of lung function, FeNO and inflammatory cytokines.

	Asthma group	Control group	*p*
FEV1%	77.26 ± 23.83	94.85 ± 10.44	<0.001
FEV1/FVC%	82.68 ± 15.88	91.49 ± 7.18	0.009
PEF%	76.27 ± 27.44	91.95 ± 15.81	0.013
MEF25%	48.85 (28.00–71.00)	64.00 (61.00–86.10)	0.004
MEF50%	59.96 ± 32.83	83.54 ± 11.92	<0.001
MEF75%	65.11 ± 31.55	89.24 ± 13.13	<0.001
MMEF75/25%	59.35 ± 31.79	80.64 ± 14.40	0.002
FeNO	25.00 (19.00–43.00)	19.00 (15.00–22.00)	0.009
IgE	215.96 (45.43–446.61)	51.72 (40.46–119.27)	0.010
EOS	170.00 (130.00–320.00)	110.00 (88.00–190.00)	0.032
IL-4	0.02 ± 0.01	0.02 ± 0.02	0.284
IL-5	0.49 (0.34–0.79)	0.63 (0.49–0.79)	0.329
IL-8	14.71 (9.59–27.13)	6.43 (5.07–10.40)	<0.001
IL-9	8.59 (3.09–14.75)	10.14 (6.32–17.24)	0.342
IL-13	1.31 ± 0.88	1.65 ± 1.12	0.245
IL-17A	3.31 (0.96–17.67)	2.04 (0.71–14.50)	0.453
IL-25	3.13 (2.17–5.26)	2.17 (1.29–3.13)	0.1389
IL-33	7.17 (3.80–12.03)	5.13 (3.80–9.24)	0.574
IFN-γ	3.08 (2.41–5.71)	3.76 (3.31–5.63)	0.283

### Total RNA, Sequencing Library Quality Inspection Results

We randomly selected six patients with asthma and three healthy subjects from the two groups (the demographic information on nine subjects were displayed in Supplementary Table S1A). The RIN values of all samples were >7.0 (Supplementary Table S1B), and the 28S/18S ratios were all >0.7, indicating that the RNA was of good integrity. The Qubit^®^ 2.0 Fluorescence quantitative analysis results showed that the library was sufficiently high quality for sequencing (Supplementary Table S2). And the ratio of each base with a quality of more than 20 (Q20) was ≥85%, and all samples met the quality control requirements (Supplementary Table S3).

### Data Processing and Genome Mapping

**FIGURE 1:**
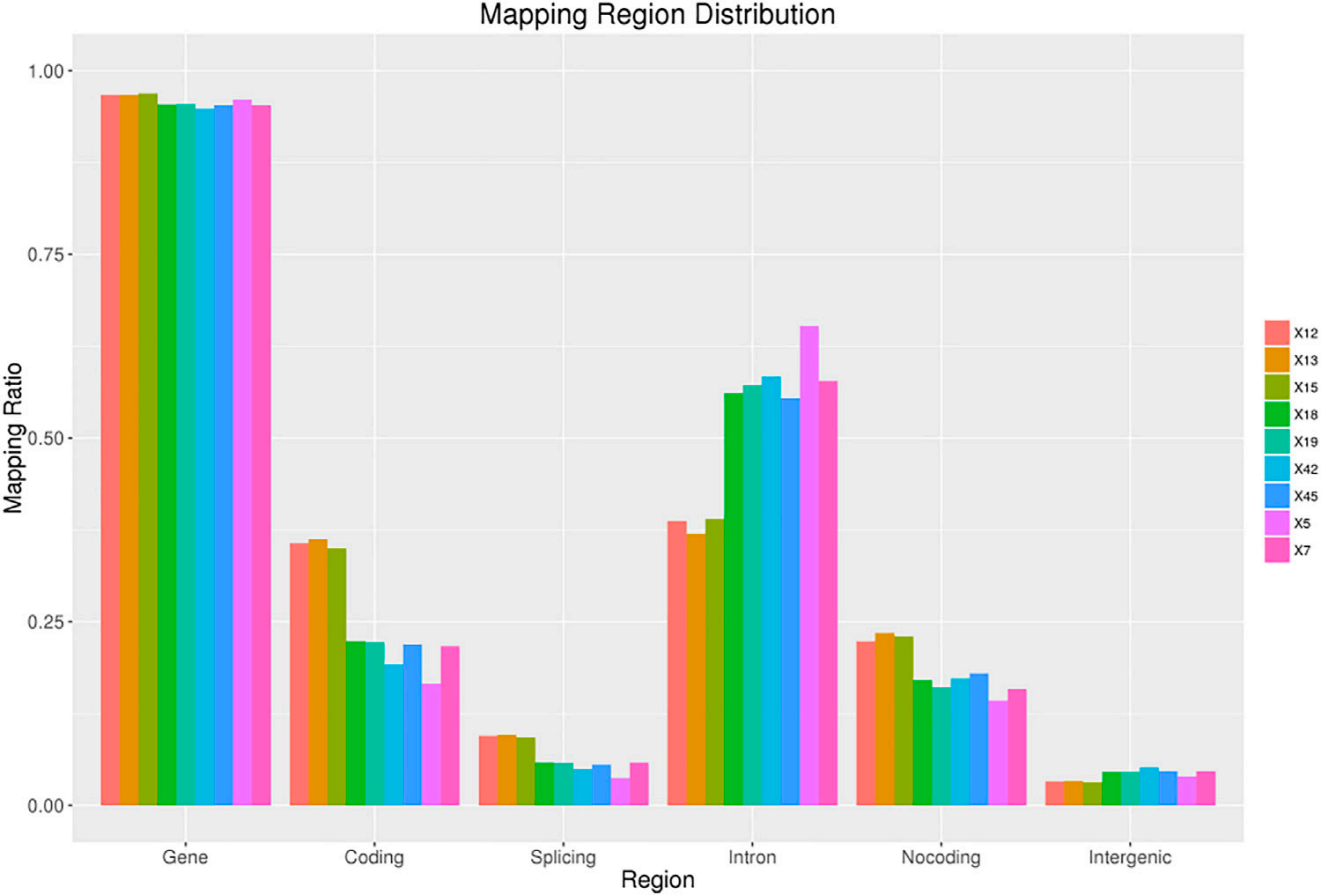
Regions distribution. The ratio of measured reads aligned to gene, coding, splicing, intron and non-coding region. Non-coding region includes statistics of total non-coding regions such as 5′ UTR, 3′ UTR, and non-coding RNA regions.

**FIGURE 2:**
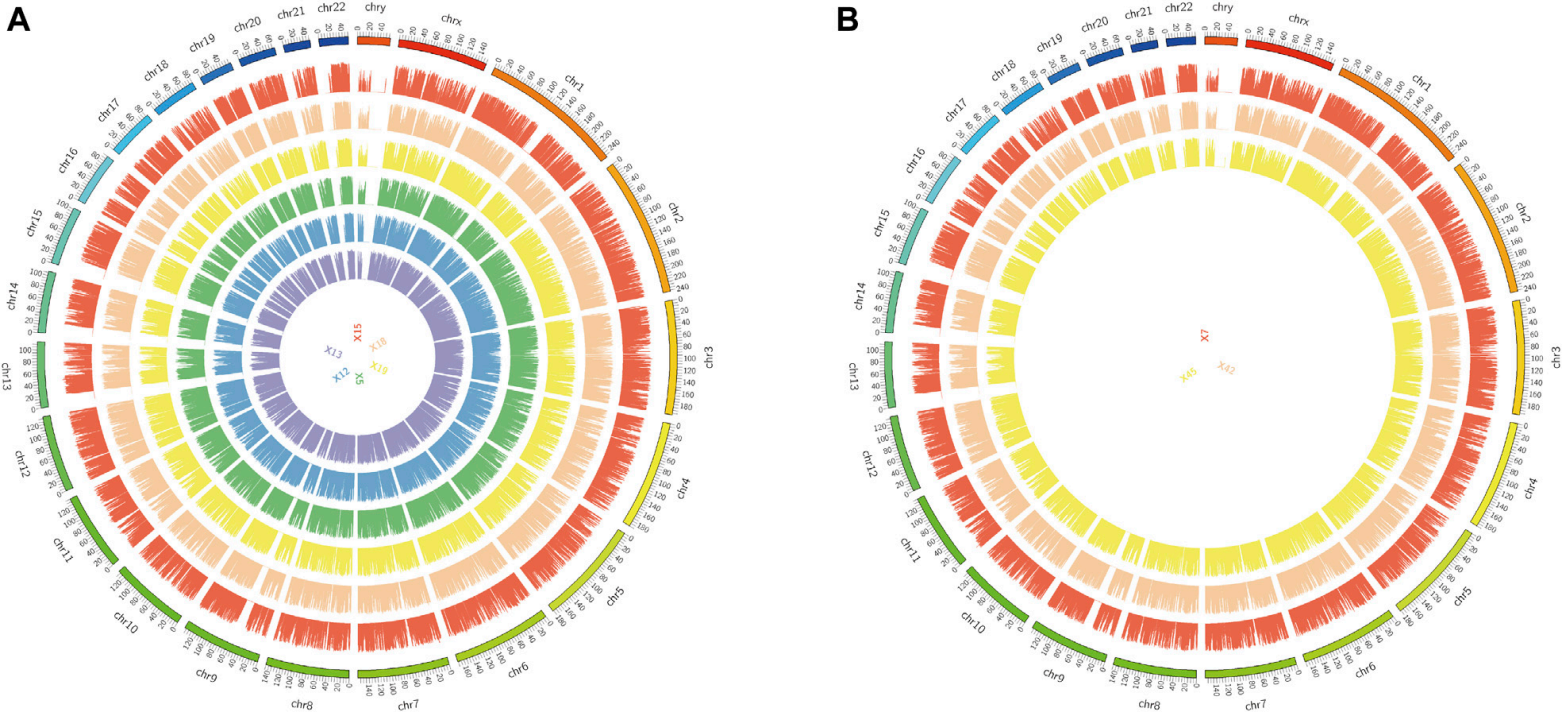
Genome coverage map. **(A)**: Genomic coverage map of asthma samples 5, 12, 13, 15, 18 and 19. **(B)**: Genomic coverage map of healthy samples 7, 42 and 45. The outermost circle in the figure is the Genome, and each circle inside represents the chromosome coverage of a sample.

The original sequences (raw reads) were filtered to obtain the effective sequences (clean reads) that could be used for data analysis (Table 4). We compared the sequences obtained with that of the reference sequence (Table 5 and Figure 1). The genome coverage distribution of each sample is shown in Figure 2, where the outermost circle is the genome, and each circle represents the chromosome coverage of a sample.

**TABLE 4:** Data processing results.

No.	Raw reads	Clean reads	Clean ratio (%)	rRNA ratio (%)	No. rRNA pair
5	78,842,400	74,321,932	94.27	0.19	73,054,476
12	82,876,584	78,243,705	94.41	0.26	76,889,770
13	77,533,108	73,731,201	95.10	0.28	72,449,512
15	71,154,652	67,801,944	95.29	0.31	66,603,148
18	70,679,030	66,843,822	94.57	0.23	65,685,676
19	67,079,192	61,883,695	92.25	0.02	60,944,216
7	74,113,180	70,950,110	95.73	0.66	69,450,354
42	79,563,670	75,110,559	94.40	0.00	74,023,280
45	77,823,786	73,605,030	94.58	0.26	72,280,382

Note: Clean ratio, (Clean reads/Raw reads) %; rRNA, ratio, [(Clean reads - rRNA, trimed)/Clean reads]%.

**TABLE 5:** Genome mapping results.

No.	All reads	Mapped reads	Mapped paired reads	Mapped unique reads	Mapping ratio (%)
5	74,023,280	72,850,058	72,290,900	72,681,496	98.42
12	73,054,476	71,617,326	70,849,638	71,362,228	98.03
13	76,889,770	75,410,004	74,587,740	75,131,921	98.08
15	72,449,512	71,065,744	70,289,274	70,815,998	98.09
18	66,603,148	64,285,423	63,711,042	64,123,082	96.52
19	65,685,676	62,310,147	61,689,166	62,133,069	94.86
7	72,280,382	69,060,901	68,400,236	68,868,720	95.55
42	60,944,216	59,382,248	58,871,286	59,213,248	97.44
45	69,450,354	67,473,749	66,879,836	67,310,181	97.15

Note: Mapping ratio, Mapped reads/All reads; Mapped unique reads: Reads that match only one position in the reference Genome.

### Correlation Analysis

**FIGURE 3:**
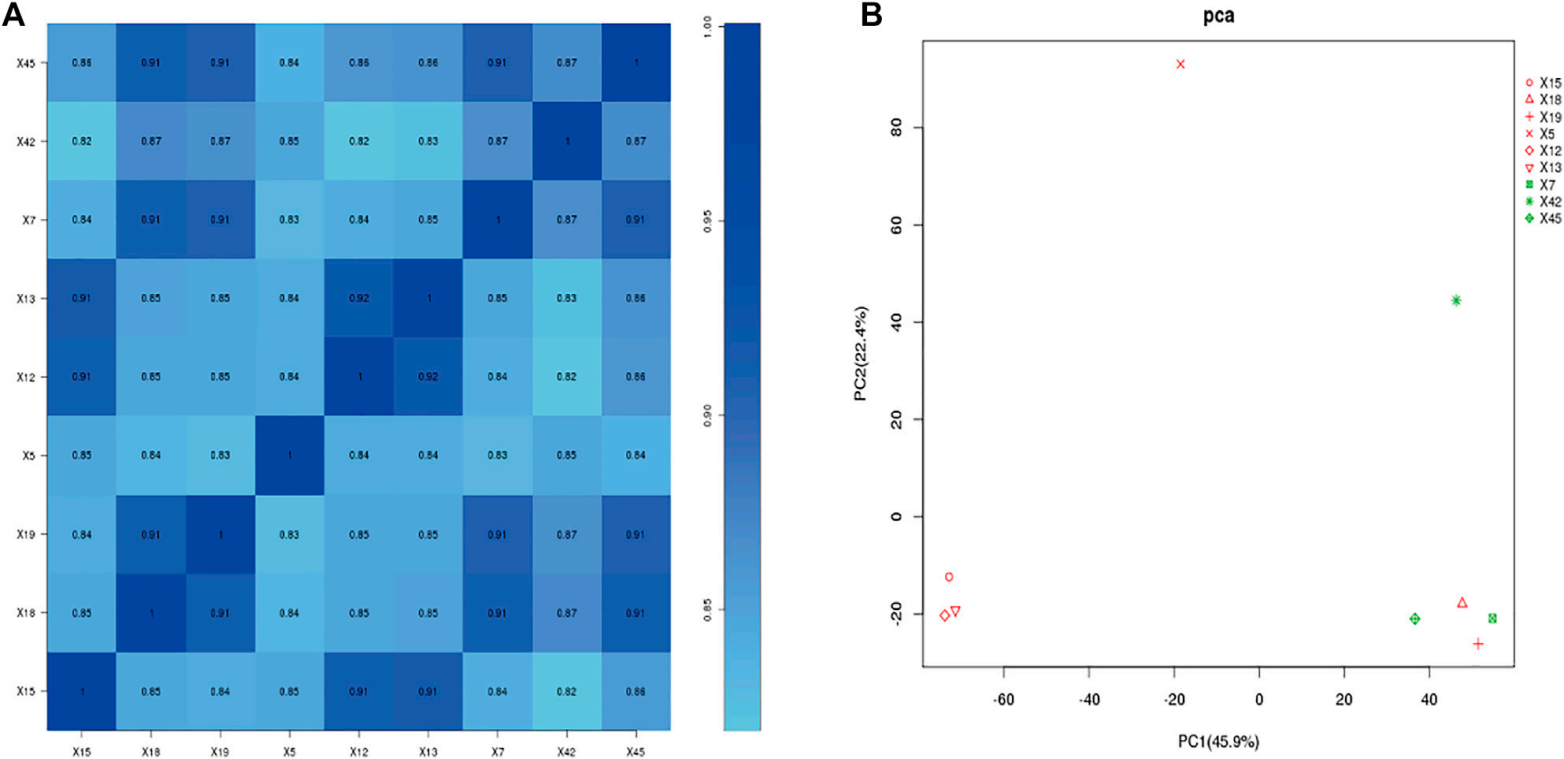
Gene expression correlation analysis. **(A)**: Correlation coefficient of each sample. **(B)**: principal component analysis. X5, X12, X13, X15: Asthma group. X7, X42, X45: Control group. Red: asthma group; Green: Control group (In PCA plot).

We performed correlation co-efficient analysis and principal component analysis (PCA) using the FPKM expression data. According to the expression correlation heat map, samples 18 and 19 of the asthma group were highly similar to samples 7 and 45 of the healthy control group, and the correlation coefficients were all >0.9. Other samples in the asthma group (5, 12, 13, and 15) and the healthy control group (7, 42, 45) had good intragroup similarity (Figure 3A). According to PCA, samples 7, 42, and 45 of the healthy control group had high similarity. Samples 5, 12, 13, and 15 in the asthma group clustered together, while samples 18 and 19 were clear outliers (Figure 3B). To ensure the accuracy and reliability of the analysis results, samples 18 and 19 were therefore excluded, and four samples from the asthma group (5, 12, 13, and 15) and three samples from the healthy control group (7, 42, 45) were selected for follow-up analysis.

### Analysis of Gene Expression in the Peripheral Blood

**FIGURE 4:**
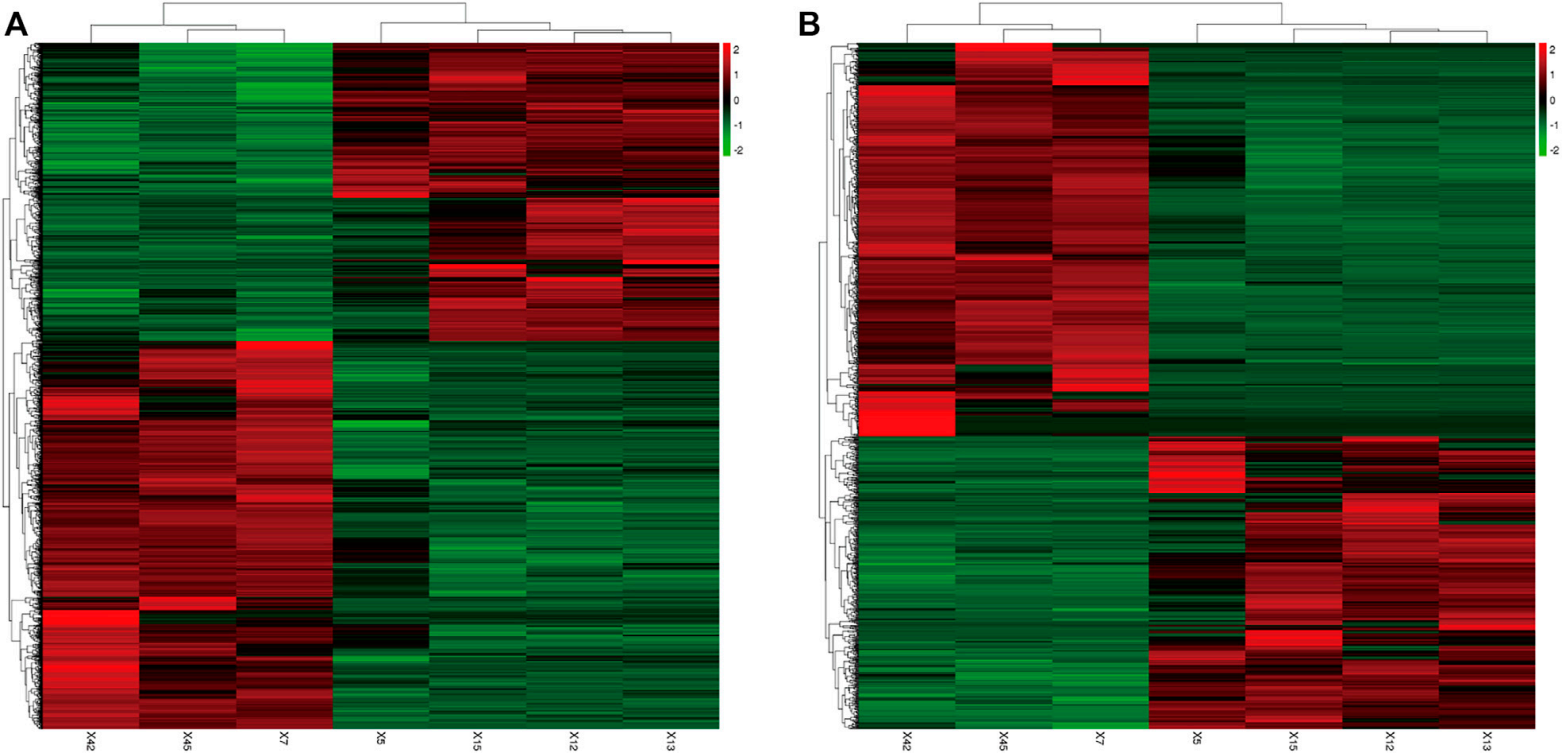
Differential Gene Expressions (Heat map). **(A)**: Differentially expressed mRNAs. **(B)**: Differentially expressed lncRNAs. X5, X12, X13, X15: Asthma group. X7, X42, X45: Control group. Green: down-regulated; Red: up-regulated.

**FIGURE 5:**
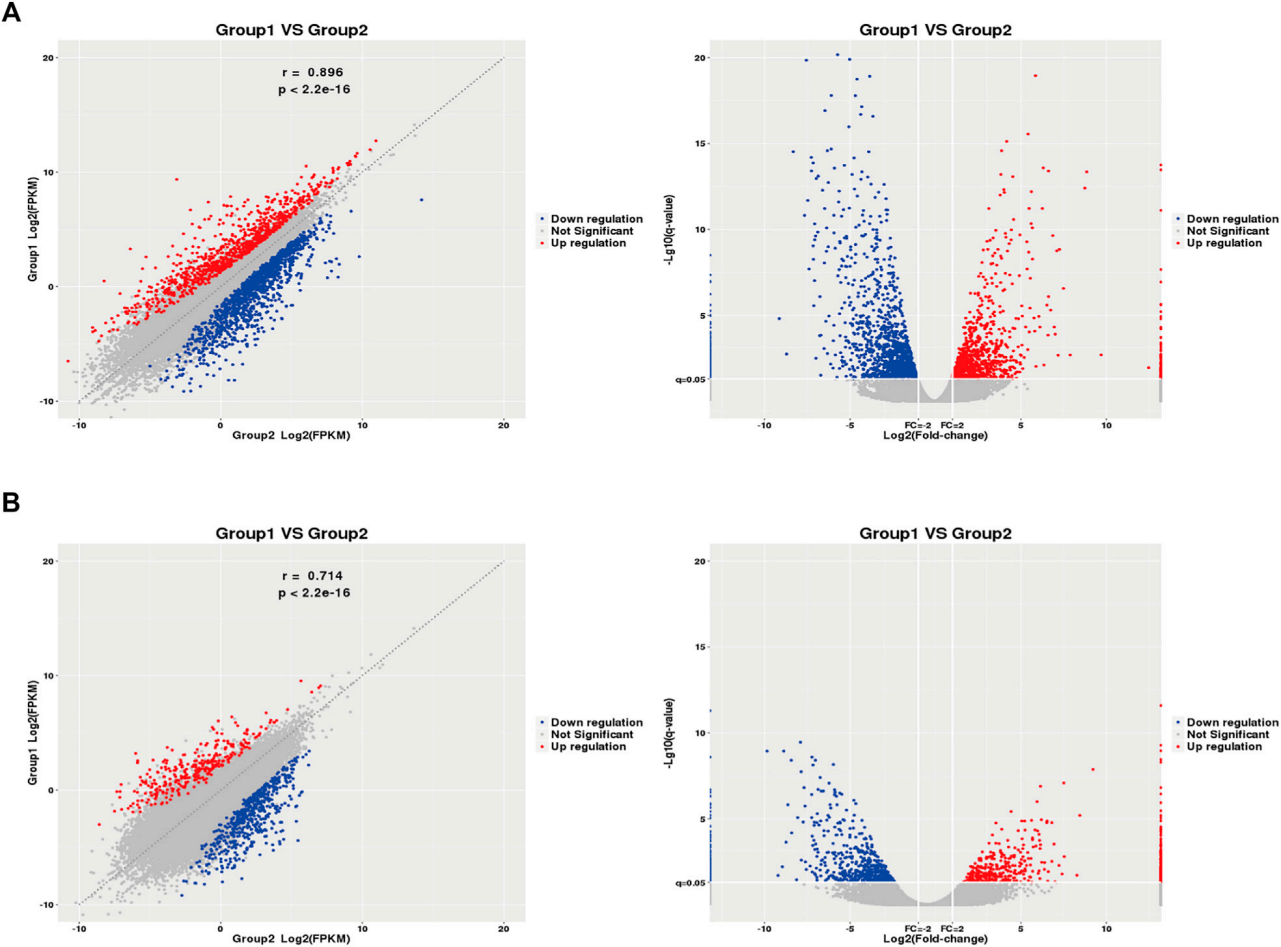
D**i**fferential Gene Expressions (scatter and volcano plots). **(A)**: Differentially expressed mRNAs. **(B)**: Differentially expressed lncRNAs. X5, X12, X13, X15: Asthma group. X7, X42, X45: Control group. Blue: down-regulated; Red: up-regulated. FPKM: Fragments Per kilobase of exon model per Million mapped reads.

2,290 mRNAs (994 up-regulated and 1,296 down-regulated) and 1,137 lncRNAs (485 up-regulated and 652 down-regulated) were differentially expressed between asthma group and healthy control group. The cluster analysis was used for the similarly expressed mRNAs and lncRNAs, and the heatmaps were drawn. As shown in Figure 4, red color indicated that the genes were up-regulated, while green color was down-regulated. The darker the color was, the higher the multiples of up-regulation or down-regulation it indicated. In addition, the top 50 up and down-regulated lncRNAs (Supplementary Tables S4, S5) as well as top 50 mRNAs (Supplementary Tables S6, S7) were screened out. Scatter plots and volcano plots were generated to display the differences between the two groups (Figure 5).

### Comparative Analysis of Differentially Expressed lncRNAs and mRNAs

**FIGURE 6:**
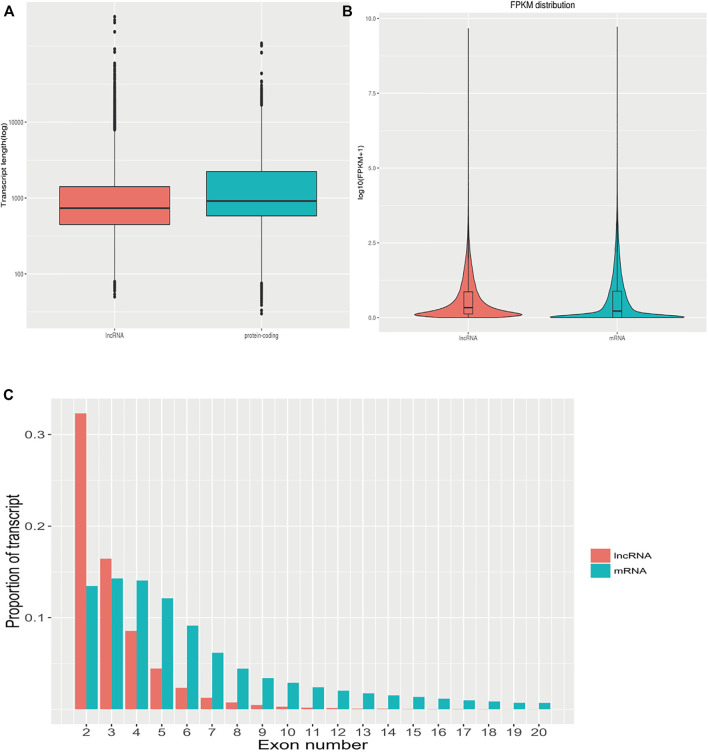
The characteristics of differentially expressed lncRNAs and mRNAs. **(A)**: Comparison of the transcript length of lncRNAs and mRNAs. **(B)**: Comparison of lncRNAs and mRNAs expression levels. **(C)**: Comparison of the number of exons between lncRNAs and mRNAs.

The transcript length, number of exons, and expression levels of differentially expressed lncRNAs and mRNAs were compared and analyzed. The length of the lncRNA transcripts was similar to that of mRNA. The proportion of lncRNA transcripts with ≤3 exons was significantly greater than that of mRNA. The average FPKM expression of each lncRNA and mRNA transcript was used to generate a box plot of log10 (FPKM+1). The expression levels of lncRNA were similar to those of mRNA but were more uniform in the expression range of 0–2.5 FPKM (Figure 6). This suggests that the lncRNAs identified in the present study conform to the general characteristics of lncRNAs.

### GO and KEGG Analysis of Differentially Expressed Genes

**FIGURE 7:**
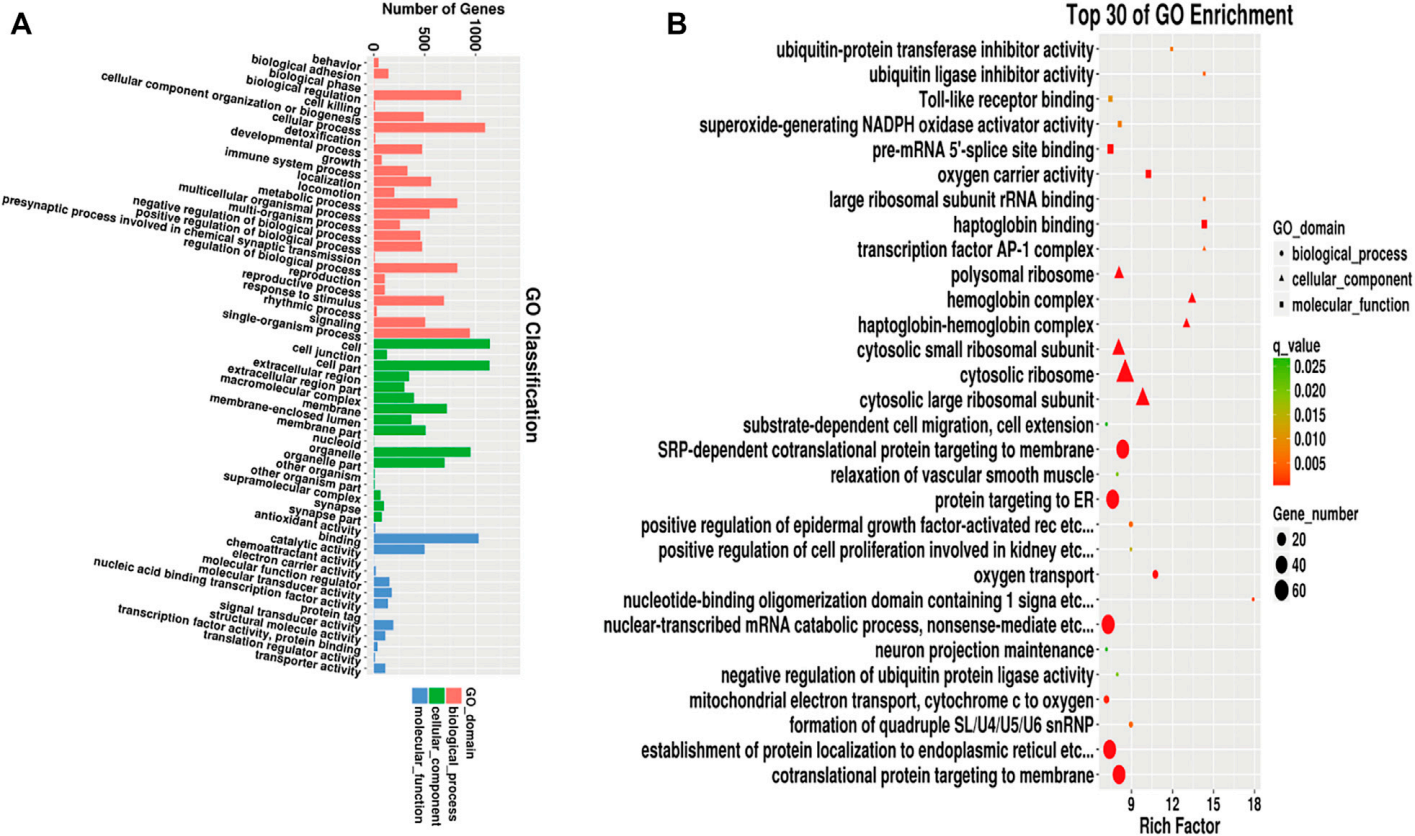
GO analysis of differentially expressed genes. **(A)**: GO function classification map of differentially expressed genes. **(B)**: Scatter plot of GO enrichment of top 30 differentially expressed genes.

We next performed GO analysis using the DEGs identified by sequencing. Of the DEGs, 2,290 mapped on to 57 functional GO terms. Among these, 26 were related to biological processes, with cellular process (GO:0009987) being the most frequent, 17 were related to cellular component, mainly cell (GO:0005623) and cell part (GO:0044464), and 14 were related to molecular functions, mainly binding (GO:0005488) (Figure 7A).

We next performed GO enrichment analysis on the differentially expressed genes and selected the top 30 significantly enriched GO entries. As shown in Figure 7B, the enriched biological processes included nuclear-transcribed mRNA catabolic process (GO:0000956), establishment of protein localization to endoplasmic reticulum (GO:0072599), endoplasmic reticulum protein targeting process (protein targeting to ER, GO:0006612), and co-translational protein targeting to the membrane (GO:0006613). The most enriched cellular component was cytosolic ribosomes (GO:0022626) (see Supplementary Table S8).

**FIGURE 8:**
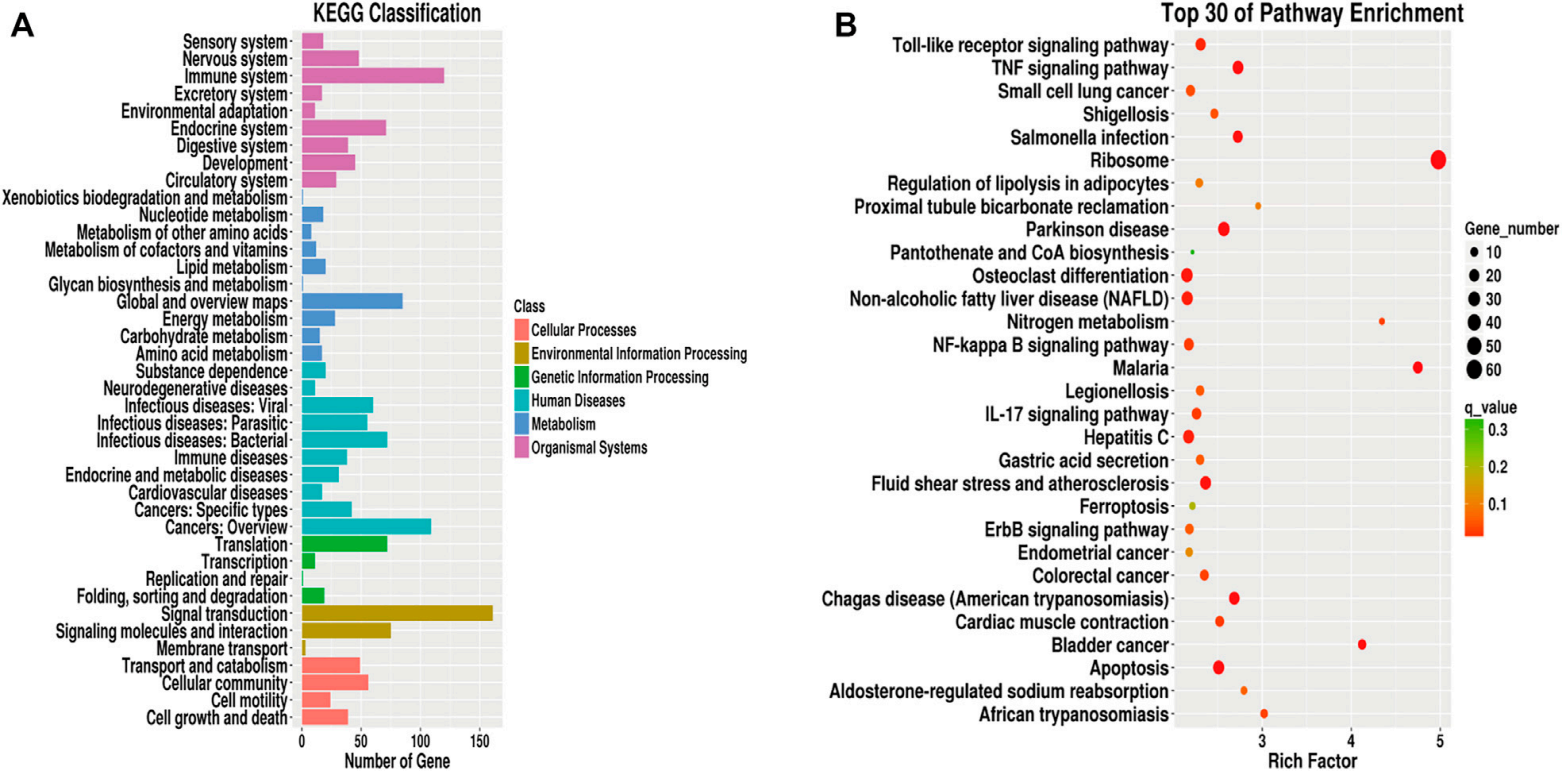
KEGG analysis of differentially expressed genes. **(A)**: KEGG pathway classification map of differentially expressed genes. **(B)**: KEGG enrichment scatter plot of differentially expressed genes.

We next performed KEGG pathway enrichment analysis on the differentially expressed genes between the two groups, and identified the top 30 enriched KEGG pathways. As shown in Figures 8A,B, pathways related to the immune system, signal transduction, translation, cancers: overview, global and overview maps, cellular community, and environmental information processing were enriched, among which signal transduction in environmental information processing were the most enriched. Among the top 30 enriched signaling pathways, the ribosome (hsa03010) contained the highest number of DEGs (see Supplementary Table S9).

### Differentially Regulated lncRNA and lncRNA-mRNA Co-expression Network

**FIGURE 9:**
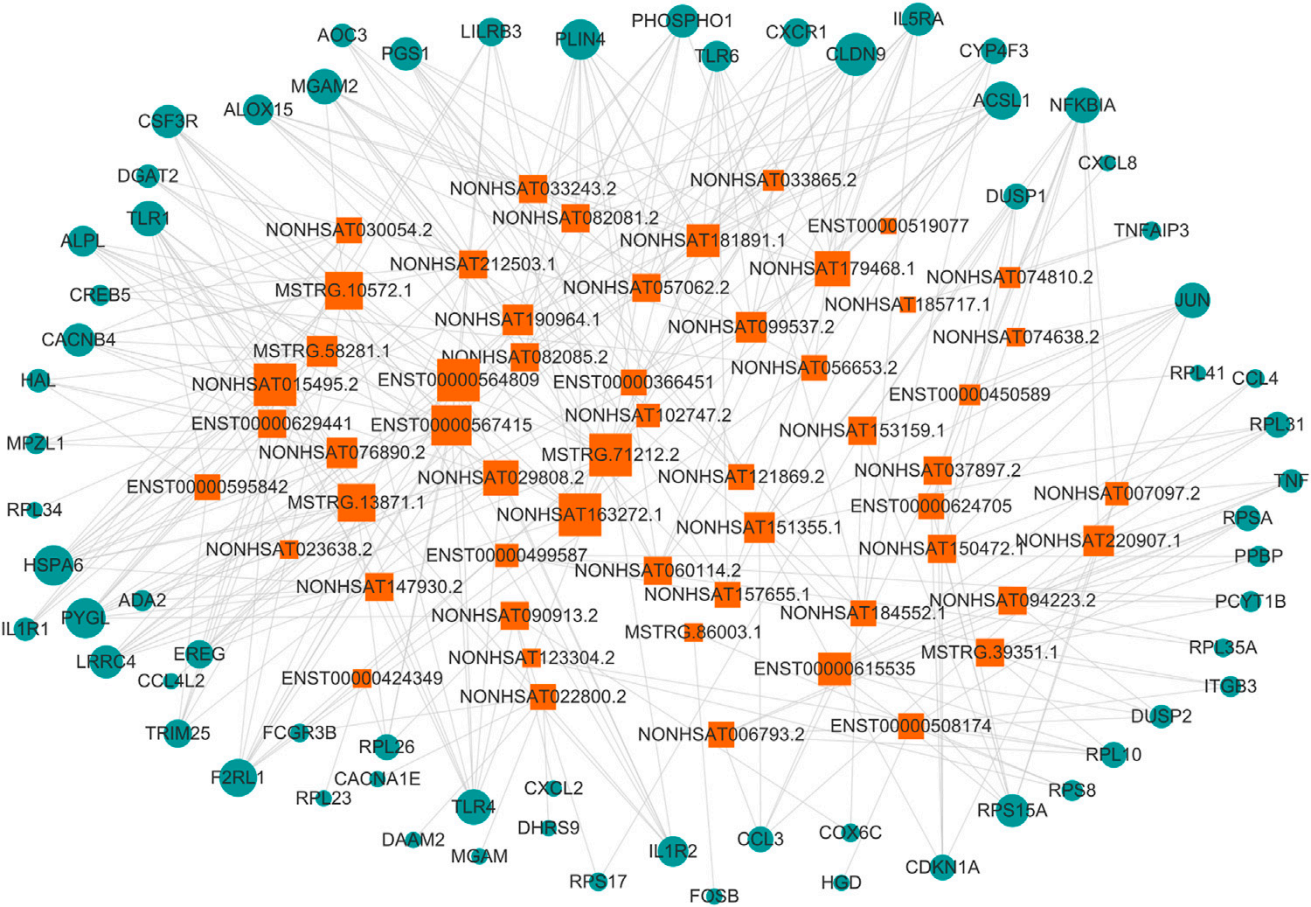
**l**ncRNA-mRNA co-expression network. The circle represents mRNA, the square represents lncRNA, and the line between the circle and the square represents the regulatory relationship between genes. The relationship between a gene and other genes in the network is represented by the “degree value”, the greater the degree value, the larger the area of the circle and the square.

We filtered the top 50 upregulated lncRNAs (Supplementary Table S4) and the top 50 downregulated lncRNAs (Supplementary Table S5) in the asthma group compared to in the healthy control group. Based on the KEGG pathway enrichment analysis, we selected differentially expressed mRNAs in 25 signaling pathways (Table 6) that were related to asthma in the literature. According to lncRNA-mRNA co-expression analysis (Figure 9), the top 10 lncRNAs in the co-expression network were selected, of which seven were down-regulated and three were up-regulated (Table 7). Among these, IDs starting with MSTRG are novel lncRNAs, IDs starting with NON are known lncRNAs in the database, and IDs starting with ENS are known lncRNAs in the Ensembl database.

**TABLE 6:** Asthma-related KEGG enrichment pathway.

Pathway_id	Pathway	Diff_Genes	Rich factor
hsa01100	Metabolic pathways	84	0.902
hsa03010	Ribosome	60	4.979
hsa04060	Cytokine-cytokine receptor interaction	46	2.016
hsa04151	PI3K-Akt signaling pathway	43	1.694
hsa04010	MAPK signaling pathway	38	1.691
hsa04062	Chemokine signaling pathway	28	1.897
hsa04210	Apoptosis	26	2.510
hsa04668	TNF signaling pathway	24	2.727
hsa00190	Oxidative phosphorylation	23	2.150
hsa04024	cAMP signaling pathway	23	1.530
hsa04621	NOD-like receptor signaling pathway	21	1.385
hsa04620	Toll-like receptor signaling pathway	20	2.308
hsa04514	Cell adhesion molecules (CAMs)	20	1.098
hsa04064	NF-kappa B signaling pathway	19	2.175
hsa04630	JAK-STAT signaling pathway	18	1.592
hsa04657	IL-17 signaling pathway	17	2.262
hsa04150	mTOR signaling pathway	16	1.343
hsa04659	Th17 cell differentiation	12	0.909
hsa04650	Natural killer cell mediated cytotoxicity	11	0.339
hsa04660	T cell receptor signaling pathway	9	1.156
hsa04662	B cell receptor signaling pathway	8	1.515
hsa03320	PPAR signaling pathway	8	1.477
hsa04750	Inflammatory mediator regulation of TRP channels	8	1.158
hsa04152	AMPK signaling pathway	8	0.916
hsa04310	Wnt signaling pathway	7	0.601

**TABLE 7:** Top 10 lncRNAs with degree value.

lncRNA_id	lncRNA_name	Up/down	degree
NONHSAT015495.2	lnc-FAS-2-2_dup1	down	12
MSTRG.71212.2	—	down	12
NONHSAT163272.1	—	down	12
ENST00000564809	lnc-KLHL36-1-1	down	12
NONHSAT181891.1	XLOC_001496	down	9
ENST00000615535	—	up	8
NONHSAT151355.1	m130127_174,513_00126_c100	up	7
NONHSAT190964.1	lnc-DSCR8-3-1_dup1	down	7
NONHSAT220907.1	m121212_111,138_00126_c100	up	7
NONHSAT076890.2	lnc-CXCR2-1-5_dup1	down	7

### Verification of Differentially Expressed lncRNAs

**FIGURE 10:**
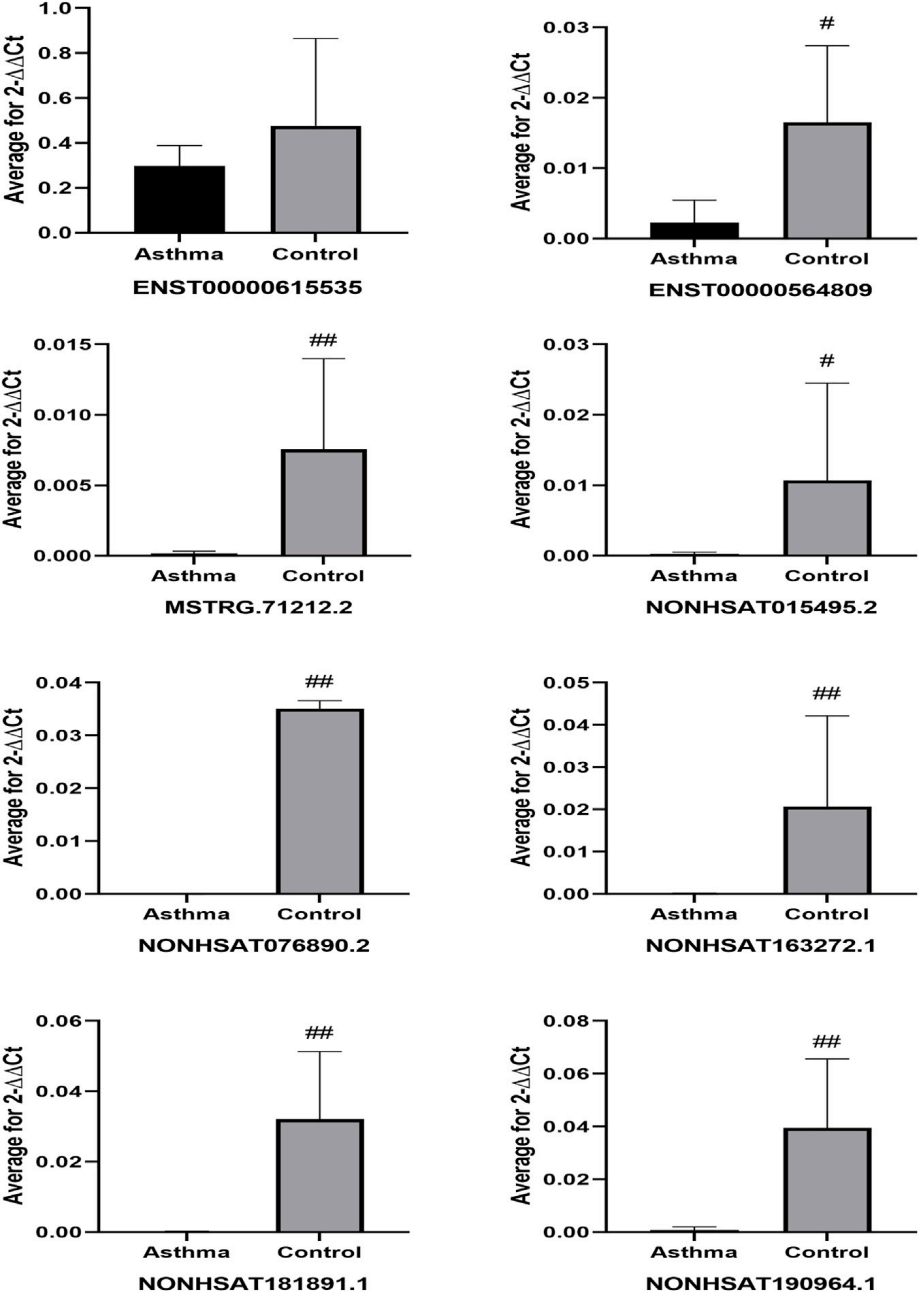
Differential expression of lncRNAs in sequencing samples. ^#^ *p* < 0.05, ^##^ *p* < 0.01 *vs* Asthma group.

We performed qPCR on peripheral blood samples from four asthma patients and three healthy subjects that were previously sequenced to verify the sequencing results. The gene sequence of lncRNA NONHSAT151355.1 overlaps completely with the mRNA sequence LAPTM5, and the gene sequence of lncRNA NONHSAT220907.1 overlaps completely with the mRNA sequence of UBQLN1, so specific primers could not be designed for these lncRNAs. Therefore, we designed specific primer sets for the remaining eight asthma-associated lncRNAs. As shown in Figure 10, the relative expression of lncRNA ENST00000615535 in the asthma group was lower than that in the healthy control group, which was inconsistent with the sequencing results, but this difference was not statistically significant (*p* = 0.535). The expression levels of the other 7 lncRNAs in the asthma group were all lower than in the healthy control group, which was consistent with the sequencing results, and the differences were statistically significant.

**FIGURE 11:**
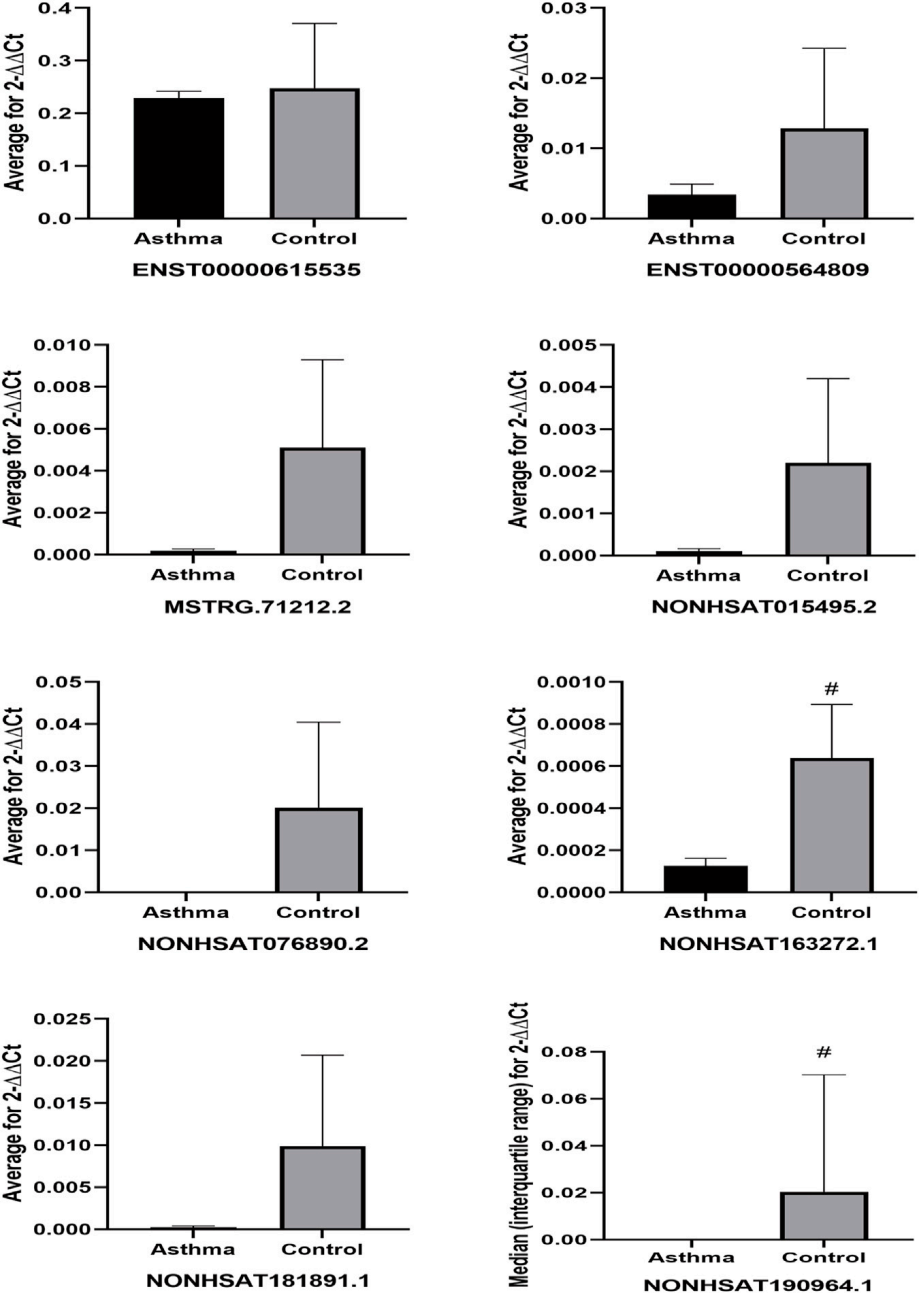
Differential expression of lncRNAs in expanding samples. #p < 0.05 vs Asthma group.

To further verify that the identified lncRNAs were an asthma-specific signature, peripheral blood samples were obtained from three asthma patients and three healthy subjects that were randomly selected from the remaining subjects that were not included in the sequencing dataset. qPCR analysis of these expanded samples revealed that the expression trends of two of the lncRNAs (NONHSAT163272.1 and NONHSAT190964.1) were consistent with the trends observed in the sequencing dataset, and these differences were statistically significant (both *p* < 0.05) (Figure 11). Although the remaining target lncRNAs had no statistically significant differences in the expanded sample set between the asthma and healthy control groups, their expression trend was consistent with those of the sequenced samples.

Next, we compared the expression of the 7 identified lncRNAs from the present study with their expression in four public databases in the Gene Expression Omnibus (GEO). Among these four databases, two of them performed lncRNA sequencing of whole blood from asthma patients (GSE106230, https://www.ncbi.nlm.nih.gov/geo/query/acc.cgi?acc=GSE106230, adults; GSE117038, https://www.ncbi.nlm.nih.gov/geo/query/acc.cgi?acc=GSE117038, adults), and the other two used microarrays to detect the expression of lncRNAs in the peripheral blood mononuclear cells (PBMCs) of asthmatic patients (GSE165934, https://www.ncbi.nlm.nih.gov/geo/query/acc.cgi?acc=GSE165934, adults; GSE143192, https://www.ncbi.nlm.nih.gov/geo/query/acc.cgi?acc=GSE143192, children).

**FIGURE 12:**
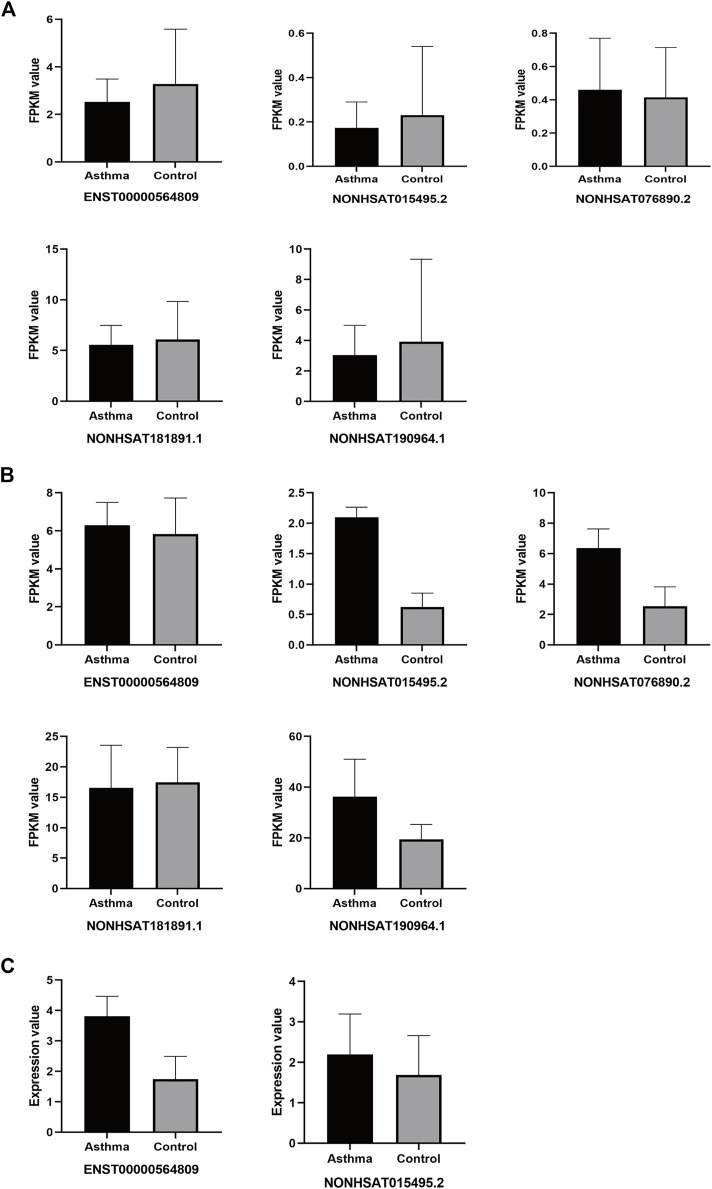
Differential expression of target lncRNAs in public databases. **(A)**: Each target of differential lncRNAs in the GSE106230 database; **(B)**: Each target of differential lncRNAs in the GSE117038 database; **(C)**: Each target of differential lncRNAs in the GSE143192 database.

For the original data from GSE106230 and GSE117038, we performed differential lncRNA analysis (Supplementary Figure S1) and then matched these results with the 7 lncRNAs from the present study. For those lncRNAs with no matches in the differential lncRNA analysis results, we matched them to the entire GSE106230 and GSE117038 database. For the original data from GSE165934 and GSE143192, due to the different samples and detection methods used, we directly performed the comparison. As shown in Figure 12, in the GSE106230 and GSE117038 datasets contained five of the targeting differentially expressed lncRNAs from the present study (ENST00000564809, NONHSAT015495.2, NONHSAT076890.2, NONHSAT181891.1, and NONHSAT190964.1). In the GSE106230 database, the FPKM values for the lncRNAs ENST00000564809, NONHSAT015495.2, NONHSAT181891.1, and NONHSAT190964.1 showed a downward trend compared with the healthy group, which is consistent with our results, although the differences between the case and control groups were not statistically significant (Figure 12A). In the GSE117038 database, we excluded samples from patients with neutrophilic asthma (the patients we included were eosinophil-dominated). As shown in Figure 12B, compared with the healthy group, these five lncRNAs were not statistically different in the asthmatic group in this dataset. Four of the lncRNAs (ENST00000564809, NONHSAT015495.2, NONHSAT076890.2, and NONHSAT190964.1) were slightly upregulated in the asthma group compared with in healthy subjects, which contradicts our results. There were no matches between the seven identified lncRNAs from the present study in the GSE165934 dataset. In the GSE143192 database, the FPKM values of ENST00000564809 and NONHSAT015495.2 were slightly increased in children with asthma compared with in healthy children (Figure 12C).

## Discussion

Asthma is a chronic inflammatory disease of the lower respiratory tract that is characterized by reversible airflow obstruction and airway remodeling (Bousquet et al., 2000). Early research on the mechanisms of asthma mainly focused on T helper (Th) cells. It is generally thought that a Th1/Th2 imbalance (Th1 inhibition and Th2 hyperfunction) is the main cause of airway hyperresponsiveness and chronic inflammation in asthma (Ngoc et al., 2005). Recent research has advanced our understanding of the pathogenesis of asthma, and suggests that a Treg/Th17 imbalance is as important as a Th1/Th2 imbalance, and is closely related to the progression and severity of asthma (Wang et al., 2018). However, this cannot fully explain the pathogenesis of asthma. For example, under certain circumstances, the enhancement of Th1 activity can aggravate asthma symptoms, rather than acting as a protective factor (Zou et al., 2018). With the development of advanced technology for studying epigenetics and transcriptomics in recent years, there is growing evidence that non-coding RNAs are involved in the process of asthma pathology, and play a crucial role in pre- and post-transcriptional regulation (Ghafouri-Fard et al., 2020), such as lncRNAs can regulate the proliferation, differentiation, and induction of immune/structural cells, and are closely related to asthma (Austin et al., 2017; Zhu et al., 2019; Liu et al., 2020).

Although there have been clinical trials to detect the differential expression of lncRNAs in the peripheral blood of patients with asthma (Gál et al., 2020; Zheng et al., 2020), there are many lncRNAs that have not been annotated. Finding other lncRNAs that may play a key role in asthma is therefore important, and may contribute to a better understanding and treatment of asthma. Therefore, in the present study, we used second-generation high-throughput sequencing technology to perform whole-transcriptome sequencing in asthma patients and healthy subjects, and identified the differentially expressed mRNAs and lncRNAs. We identified a total of 2,290 differentially expressed mRNAs and 1,137 differentially expressed lncRNAs in asthma patients compared with in healthy subjects. Among these, there were 994 upregulated and 1,296 downregulated mRNAs, and 485 upregulated and 652 downregulated differential lncRNAs. We conducted a comparative analysis of the identified lncRNAs and mRNAs to further verify that the lncRNAs were lncRNAs. Then, we performed GO and KEGG functional analysis of the differentially expressed genes. The GO and KEGG enrichment results showed that the cytosolic ribosome (GO:0022626) and ribosome (hsa03010) were highly enriched in the DEGs. This may be because mRNA carries the code from the DNA in the nucleus to the ribosome in the cytoplasm, and protein synthesis is often regulated by structured mRNAs that interact with ribosomes (Boehringer and Ban, 2007). For example, gene expression can be regulated by structural elements in the 5′ untranslated region of mRNA at the initial level of protein biosynthesis, and these folded mRNA fragments may bind to ribosomes, preventing translation (Marzi et al., 2007). We selected the top 50 up-regulated and top 50 down-regulated lncRNAs and identified the top 25 pathways related to asthma. Following co-expression analysis, we generated a lncRNA-mRNA co-expression network and selected the top 10 differentially expressed lncRNAs according to the descending order of degree value. Among these 10 differentially expressed lncRNAs, NONHSAT015495.2, MSTRG.71212.2, NONHSAT163272.1, ENST00000564809, NONHSAT181891.1, NONHSAT190964.1, and NONHSAT076890.2, were downregulated in asthma patients, and ENST00000615535, NONHSAT151355.1, and NONHSAT220907.1 were upregulated in asthma patients. We used qPCR to verify the sequencing results for these 10 lncRNAs. Except for the decreased expression of ENST00000615535 in asthma patients, which was inconsistent with the sequencing results, the expression of the remaining seven lncRNAs (NONHSAT015495.2, MSTRG.71212.2, NONHSAT163272.1, ENST00000564809, NONHSAT181891.1, NONHSAT190964.1, and NONHSAT076890.2) was relatively lower in asthma patients than in healthy subjects, and thus confirmed the sequencing results. To verify that these lncRNAs are an asthma-specific signature, we measured their expression by qPCR in new subjects and cases randomly selected from the remaining samples that were not sequenced, and the expression of two of the lncRNAs (NONHSAT163272.1 and NONHSAT190964.1) were consistent with the sequencing dataset.

In addition, we investigated their expression in datasets from the GEO database. The FPKM value trends of four of the differentially expressed lncRNAs identified in the present study (ENST00000564809, NONHSAT015495.2, NONHSAT181891.1, and NONHSAT190964.1) in the GSE106230 dataset were consistent with our results, although the differences were not statistically significant in that dataset. However, the FPKM value trends of the four differentially expressed lncRNAs (ENST00000564809, NONHSAT015495.2, NONHSAT076890.2, NONHSAT190964.1) in the GSE117038 database, and two lncRNAs (ENST00000564809, NONHSAT015495.2) in the GSE143192 database disagreed with the results of the present study. It is worth noting that samples used in the GSE106230 and GSE117038 databases (whole blood samples from adults), and the PBMC samples used in the GSE143192 database (children) did not match the sample type used in the present study, perhaps limiting the utility of this comparison.

The present study had some limitations. As shown in Table 3, there were no statistically significant differences observed for Th2 cytokines, including IL-4, IL-5, and IL-13, between the asthma and healthy control subjects in the present study. This may be due to the choice of peripheral blood as detection samples, and the regular treatment of low dose ICS/LABA for asthmatic patients before the recruitment. It is agreed that the main pathology of asthma patients is in the airways, and Bronchoalveolar Lavage Fluid (BALF) may therefore be a better sample type for the detection of asthma-derived inflammatory factors. However, fiberoptic bronchoscopy is an invasive procedure and not routinely performed in asthma patients, which made the collection of BALF difficult. As venous blood was easy to collect and had little harm to participants, it was used to detect the cytokine levels instead; on the other hand, the regular use of ICS/LABA in asthma patients might to some degree alleviate the inflammatory status so that rare differences of inflammatory cytokines were found between asthma patients and healthy subjects. Due to the small sample size of the present study, the proposed asthma-specific marker lncRNAs could not be confirmed as biomarkers for asthma. To address this, multicentered clinical study with a larger sample size should be undertaken. In addition, the composition of lncRNAs present in the peripheral blood may be affected by many factors, and we did not take the atopic status and some allergy-related co-morbidities into consideration during the participants recruitment, and to some degree this negligence potentially affect the gene expressions in PBMCs in whole blood. Animal models could be used to measure their expression in the lung tissue and BALF specifically, which may provide more compelling evidence that they are directly related to the pathology of asthma. The regulatory effect of these lncRNAs in immune/structural cells should also be confirmed by *in vitro* studies, which could also provide much-needed functional annotation for these potentially important lncRNAs.

In conclusion, we have identified a panel of putative asthma-associated lncRNAs (NONHSAT015495.2, MSTRG.71212.2, NONHSAT163272.1, ENST00000564809, NONHSAT181891.1, NONHSAT190964.1, and NONHSAT076890.2), which may play a role in the pathogenesis of asthma, and may be useful as diagnostic or prognostic biomarkers in the clinical setting.

## Data Availability

The datasets presented in this study can be found in online repositories. https://www.ncbi.nlm.nih.gov/geo/query/acc.cgi?acc=GSE195599.
