# Supplementary material for: Screening and Verification of Differentially Expressed Long Non-coding RNAs in the Peripheral Blood of Patients With asthma
Source: Front Pharmacol. 2022 Feb 22;13:834009. doi: 10.3389/fphar.2022.834009 (PMC8902465; doi:10.3389/fphar.2022.834009)
Supplement: Supplementary file 1 [file DataSheet1.docx]

**Supplementary Tables and Figures**

**Supplementary Table 1A．Demographic information on nine subjects used in sequencing**

| **Group** | **Sample number** | **Sex Male=1, Female=2** | **Age** | **BMI** | **FEV1%** | **FEV1/FVC%** | **PEF%** | **PEF25%** | **PEF50%** | **PEF75%** | **MMEF75/25%** | **FeNO** | **IgE** | **EOS** | **EOS（%）** | **IFN-γ** | **IL-4** | **IL-5** | **IL-8** | **IL-9** | **IL-13** | **IL-17a** | **IL-25** | **IL-33** |
| --- | --- | --- | --- | --- | --- | --- | --- | --- | --- | --- | --- | --- | --- | --- | --- | --- | --- | --- | --- | --- | --- | --- | --- | --- |
| **A** | 5 | 2 | 49 | 25.24 | 64.00 | 87.00 | 67.00 | 26.00 | 41.00 | 68.00 | 41.00 | 94 | 246.88 | 360 | 7.2 | 2.41 | 0.32 | 0.79 | 18.01 | 5.26 | 4.17 | 0.96 | 4.17 | 10.63 |
| **A** | 12 | 2 | 37 | 23.50 | 113.20 | 81.05 | 111.10 | 59.70 | 75.40 | 98.70 | 75.60 | 89 | 674.40 | 506 | 7.8 | 0.34 | 0.26 | 0.49 | 5.81 | 9.11 | 2.79 | 1.23 | 7.61 | 10.63 |
| **A** | 13 | 1 | 36 | 25.46 | 96.60 | 80.68 | 114.10 | 67.90 | 76.20 | 82.00 | 84.20 | 29 | 1353.20 | 176 | 2.9 | 5.71 | 0.26 | 0.49 | 5.11 | 9.46 | 6.26 | 17.67 | 4.33 | 2.39 |
| **A** | 15 | 2 | 38 | 20.07 | 35.00 | 54.00 | 32.00 | 18.00 | 14.00 | 15.00 | 15.00 | ~ | 548.76 | 380 | 8.7 | 2.86 | 0.32 | 0.79 | 5.11 | 9.80 | 3.82 | 2.32 | 3.13 | 10.63 |
| **A** | 18 | 1 | 42 | 25.54 | 91.90 | 81.89 | 97.90 | 56.10 | 69.80 | 81.80 | 71.00 | 22 | 21.10 | 176 | 2.7 | 39.48 | 4.86 | 1.27 | 21.32 | 39.35 | 3.13 | 42.71 | 67.57 | 69.80 |
| **A** | 19 | 2 | 49 | 26.91 | 55.00 | 75.00 | 42.00 | 32.00 | 22.00 | 22.00 | 22.00 | ~ | 74.16 | 190 | 2.8 | 4.68 | 7.50 | 0.79 | 48.50 | 20.23 | 4.51 | 37.52 | 17.02 | 13.44 |
| **N** | 7 | 2 | 48 | 23.56 | 115.40 | 98.44 | 89.60 | 125.20 | 91.50 | 96.50 | 103.80 | 15 | 77.41 | 132 | 1.7 | 3.31 | 0.32 | 0.34 | 161.08 | 6.32 | 3.82 | 2.04 | 6.41 | 17.70 |
| **N** | 42 | 2 | 46 | 20.96 | 93.60 | 89.09 | 72.80 | 80.10 | 83.70 | 77.10 | 85.40 | 8 | 49.82 | 66 | 1.5 | 4.68 | 0.32 | 0.49 | ~ | 23.85 | 3.48 | 0.96 | 1.29 | 5.13 |
| **N** | 45 | 1 | 38 | 20.55 | 82.00 | 103.00 | 78.00 | 77.00 | 84.00 | 84.00 | 90.00 | 22 | 68.97 | 90 | 2.1 | 2.41 | 0.20 | 0.63 | 3.70 | 5.26 | 2.09 | 0.22 | 2.17 | 6.49 |

| **Supplementary Table 1B. Total RNA quality results** | | | | | | | |
| --- | --- | --- | --- | --- | --- | --- | --- |
| Serial number | Sample number | Concentration （ng/μL） | Volume （μL） | Total （μg） | A260/ A280 | RIN | 28S/18S |
| 001 | 5 | 387 | 12 | 4.64 | 2.07 | 7.4 | 1.0 |
| 002 | 12 | 65.3 | 12 | 0.78 | 2.07 | 7.5 | 1.8 |
| 003 | 13 | 113 | 12 | 1.36 | 2.11 | 8.5 | 1.8 |
| 004 | 15 | 283 | 12 | 3.40 | 2.03 | 8.7 | 1.9 |
| 005 | 18 | 98.6 | 70 | 6.90 | 2.25 | 8.4 | 1.8 |
| 006 | 19 | 125 | 70 | 8.75 | 2.23 | 7.7 | 1.7 |
| 007 | 7 | 68.5 | 70 | 4.80 | 2.24 | 7.5 | 1.4 |
| 008 | 42 | 54.3 | 70 | 3.80 | 2.26 | 8.3 | 1.7 |
| 009 | 45 | 61.2 | 70 | 4.28 | 2.26 | 8.0 | 1.2 |

| **Supplementary Table 2. Qubit^®^ 2.0** **Fluorescence quantitative analysis results** | | | | |  |
| --- | --- | --- | --- | --- | --- |
| Serial number | Sample number | Index number | Concentration (ng/μL) | Main peak length (bp) |  |
| 001 | 5 | V17 | 24.6 | 380 | |
| 002 | 12 | V18 | 18.4 | 388 | |
| 003 | 13 | V19 | 24.2 | 384 | |
| 004 | 15 | V20 | 32.8 | 382 | |
| 005 | 18 | V21 | 41.6 | 378 | |
| 006 | 19 | V22 | 38.6 | 379 | |
| 007 | 7 | V23 | 32.4 | 376 | |
| 008 | 42 | V24 | 5.7 | 375 | |
| 009 | 45 | V25 | 36.2 | 380 | |

| **Supplementary Table 3.** **Sequencing data quality control results** | | | | | |  |
| --- | --- | --- | --- | --- | --- | --- |
| Serial number | Sample number | Sequence type | Base orientation | Data volume （G） | Q20 ratio（%） |  |
| 001 | 5 | mRNA | Forward/Reverse | 10.67 | 97.99% | |
| 002 | 12 | mRNA | Forward/Reverse | 10.60 | 97.99% | |
| 003 | 13 | mRNA | Forward/Reverse | 10.06 | 98.17% | |
| 004 | 15 | mRNA | Forward/Reverse | 11.12 | 97.95% | |
| 005 | 18 | mRNA | Forward/Reverse | 11.93 | 98.16% | |
| 006 | 19 | mRNA | Forward/Reverse | 11.67 | 97.96% | |
| 007 | 7 | mRNA | Forward/Reverse | 11.83 | 98.01% | |
| 008 | 42 | mRNA | Forward/Reverse | 12.43 | 98.03% | |
| 009 | 45 | mRNA | Forward/Reverse | 11.63 | 98.04% | |
| Note: Q20: bases of Q≥20/all bases of sequencing | | | | | | |

Supplementary Table 4. The top 50 up-regulated lncRNAs in asthma patients

| **lncRNA_id** | **\|log_2_FC\|** | **q-value** | **lncRNA_id** | **\|log_2_FC\|** | **q-value** |
| --- | --- | --- | --- | --- | --- |
| NONHSAT149530.1 | 6.571 | 0.043 | NONHSAT175913.1 | 4.083 | 0.003 |
| ENST00000584923 | 6.213 | 0.003 | NONHSAT176186.1 | 4.081 | 0.023 |
| NONHSAT074810.2 | 6.140 | ＜0.001 | NONHSAT074638.2 | 4.015 | 0.001 |
| NONHSAT094312.2 | 6.107 | 0.030 | NONHSAT150472.1 | 3.946 | 0.006 |
| NONHSAT193640.1 | 5.823 | ＜0.001 | NONHSAT183160.1 | 3.897 | 0.001 |
| ENST00000624705 | 5.620 | ＜0.001 | NONHSAT026887.2 | 3.883 | 0.006 |
| NONHSAT122608.2 | 5.436 | 0.040 | ENST00000424349 | 3.878 | 0.001 |
| NONHSAT119402.2 | 5.257 | 0.011 | ENST00000459748 | 3.875 | ＜0.001 |
| NONHSAT153159.1 | 5.218 | ＜0.001 | ENST00000624094 | 3.869 | 0.001 |
| ENST00000435411 | 5.148 | ＜0.001 | NONHSAT042403.2 | 3.869 | 0.007 |
| NONHSAT185717.1 | 5.047 | ＜0.001 | NONHSAT094223.2 | 3.813 | 0.023 |
| NONHSAT198297.1 | 4.970 | 0.001 | MSTRG.85242.1 | 3.807 | 0.001 |
| NONHSAT162110.1 | 4.936 | 0.004 | ENST00000450589 | 3.799 | 0.001 |
| ENST00000615535 | 4.847 | 0.001 | ENST00000465215 | 3.723 | ＜0.001 |
| NONHSAT151355.1 | 4.842 | ＜0.001 | MSTRG.39351.1 | 3.715 | 0.008 |
| NONHSAT220907.1 | 4.644 | ＜0.001 | ENST00000513886 | 3.561 | 0.005 |
| NONHSAT006793.2 | 4.534 | 0.001 | ENST00000458797 | 3.536 | ＜0.001 |
| NONHSAT122447.2 | 4.510 | 0.005 | NONHSAT055522.2 | 3.534 | ＜0.001 |
| NONHSAT090913.2 | 4.424 | ＜0.001 | NONHSAT169787.1 | 3.517 | 0.019 |
| NONHSAT037897.2 | 4.370 | 0.002 | ENST00000499587 | 3.487 | 0.015 |
| NONHSAT148574.1 | 4.277 | 0.023 | NONHSAT007097.2 | 3.434 | 0.002 |
| NONHSAT079881.2 | 4.253 | 0.039 | NONHSAT163292.1 | 3.427 | 0.001 |
| NONHSAT123304.2 | 4.241 | 0.004 | NONHSAT033865.2 | 3.392 | 0.001 |
| ENST00000508174 | 4.225 | ＜0.001 | ENST00000448718 | 3.389 | 0.009 |
| MSTRG.86003.1 | 4.088 | 0.002 | ENST00000551432 | 3.369 | 0.017 |

| **Supplementary Table 5. The top 50 down-regulated lncRNAs in asthma patients** | | | | | |
| --- | --- | --- | --- | --- | --- |
| **lncRNA_id** | **\|log_2_FC\|** | **q-value** | **lncRNA_id** | **\|log_2_FC\|** | **q-value** |
| MSTRG.33665.10 | 8.058 | ＜0.001 | NONHSAT167321.1 | 5.565 | 0.040 |
| ENST00000602591 | 7.842 | 0.003 | MSTRG.71212.2 | 5.480 | ＜0.001 |
| NONHSAT056663.2 | 6.962 | 0.008 | ENST00000559458 | 5.463 | 0.039 |
| NONHSAT102747.2 | 6.935 | ＜0.001 | NONHSAT184743.1 | 5.463 | 0.045 |
| NONHSAT070752.2 | 6.746 | 0.010 | NONHSAT022800.2 | 5.433 | 0.003 |
| NONHSAT076890.2 | 6.742 | ＜0.001 | NONHSAT163272.1 | 5.406 | ＜0.001 |
| NONHSAT033243.2 | 6.558 | ＜0.001 | NONHSAT104991.2 | 5.390 | 0.014 |
| NONHSAT152734.1 | 6.339 | 0.001 | NONHSAT056653.2 | 5.338 | ＜0.001 |
| MSTRG.10572.1 | 6.223 | ＜0.001 | NONHSAT023638.2 | 5.212 | ＜0.001 |
| NONHSAT181891.1 | 6.122 | ＜0.001 | NONHSAT060114.2 | 5.191 | 0.001 |
| ENST00000414046 | 6.012 | 0.001 | NONHSAT120405.2 | 5.042 | 0.020 |
| NONHSAT196248.1 | 6.003 | 0.023 | NONHSAT157655.1 | 5.030 | 0.011 |
| NONHSAT147930.2 | 5.971 | ＜0.001 | NONHSAT082081.2 | 5.003 | ＜0.001 |
| ENST00000595842 | 5.903 | ＜0.001 | NONHSAT039445.2 | 4.996 | 0.025 |
| NONHSAT082085.2 | 5.900 | ＜0.001 | NONHSAT099537.2 | 4.976 | 0.001 |
| NONHSAT190964.1 | 5.876 | ＜0.001 | NONHSAT057062.2 | 4.899 | 0.002 |
| NONHSAT217441.1 | 5.862 | 0.027 | MSTRG.58281.1 | 4.840 | ＜0.001 |
| ENST00000366451 | 5.774 | ＜0.001 | MSTRG.13871.1 | 4.802 | ＜0.001 |
| NONHSAT212503.1 | 5.762 | ＜0.001 | NONHSAT029808.2 | 4.779 | ＜0.001 |
| NONHSAT121869.2 | 5.684 | 0.038 | ENST00000564809 | 4.756 | ＜0.001 |
| NONHSAT196104.1 | 5.630 | 0.037 | ENST00000519077 | 4.741 | 0.001 |
| NONHSAT104639.2 | 5.603 | 0.015 | ENST00000567415 | 4.663 | ＜0.001 |
| NONHSAT030054.2 | 5.597 | ＜0.001 | NONHSAT015495.2 | 4.660 | ＜0.001 |
| NONHSAT209478.1 | 5.581 | 0.005 | NONHSAT179468.1 | 4.659 | ＜0.001 |
| ENST00000629441 | 5.580 | ＜0.001 | NONHSAT184552.1 | 4.609 | 0.012 |

Supplementary Table 6.The top 50 up-regulated mRNAs in asthma patients

| gene id | gene name | kegg_id | locus | \|log2FC\| | Pvalue | Qvalue |
| --- | --- | --- | --- | --- | --- | --- |
| ENSG00000276085 | CCL3L1 | hsa04060\|hsa04060\|hsa05163\|hsa05163\|hsa04062\|hsa04062\|hsa05323\|hsa05323\|hsa04620\|hsa04620\|hsa05142\|hsa05142\|hsa05132\|hsa05132 | 17:36194869-36196758 | 8.266191 | 3.55E-26 | 1.20E-22 |
| ENSG00000237973 | MTCO1P12 | - | 1:631074-632616 | 7.177441 | 6.59E-05 | 0.002124 |
| ENSG00000277632 | CCL3 | hsa04060\|hsa05163\|hsa04062\|hsa05323\|hsa04620\|hsa05142\|hsa05132 | 17:36088256-36090169 | 6.888618 | 1.33E-28 | 6.74E-25 |
| ENSG00000120738 | EGR1 | hsa05166\|hsa04371\|hsa04928\|hsa04933\|hsa04912\|hsa05020 | 5:138465490-138469315 | 6.738217 | 2.01E-32 | 6.09E-28 |
| ENSG00000263934 | SNORD3A | hsa03013\|hsa03008 | 17:19188016-19188714 | 6.738036 | 5.40E-08 | 5.04E-06 |
| ENSG00000265185 | SNORD3B-1 | hsa03013\|hsa03008 | 17:19061912-19062669 | 6.615409 | 7.33E-09 | 8.20E-07 |
| ENSG00000206588 | RNU1-28P | - | 14:34556226-34556389 | 6.613937 | 5.27E-17 | 3.99E-14 |
| ENSG00000274978 | RNU11 | - | 1:28648600-28648733 | 6.587127 | 2.56E-06 | 0.000143 |
| ENSG00000200169 | RNU5D-1 | - | 1:44731055-44731170 | 6.303122 | 3.14E-17 | 2.64E-14 |
| ENSG00000124882 | EREG | hsa04151\|hsa04010\|hsa04012\|hsa05210 | 4:74365143-74388751 | 5.848874 | 5.28E-23 | 1.14E-19 |
| ENSG00000284378 | MIR1244-1 | - | 2:231713314-231713398 | 5.805 | 1.38E-06 | 8.39E-05 |
| ENSG00000199377 | RNU5F-1 | - | 1:44721786-44721902 | 5.675131 | 2.85E-11 | 5.37E-09 |
| ENSG00000169429 | CXCL8 | hsa05200\|hsa04060\|hsa04218\|hsa05163\|hsa05164\|hsa05167\|hsa04621\|hsa04062\|hsa05323\|hsa05202\|hsa04932\|hsa05161\|hsa04072\|hsa04064\|hsa04620\|hsa05142\|hsa04933\|hsa04657\|hsa05146\|hsa05132\|hsa05133\|hsa04622\|hsa05120\|hsa05134\|hsa05131\|hsa05144\|hsa05219 | 4:73740506-73743716 | 5.611662 | 1.23E-15 | 6.41E-13 |
| ENSG00000279602 | AC109326.1 | - | 17:43360041-43361361 | 5.598922 | 1.34E-13 | 4.63E-11 |
| ENSG00000206596 | RNU1-27P | - | 14:34546714-34546877 | 5.549887 | 4.84E-10 | 6.98E-08 |
| ENSG00000177606 | JUN | hsa05200\|hsa05166\|hsa05169\|hsa05168\|hsa04010\|hsa05164\|hsa05170\|hsa05203\|hsa05167\|hsa04621\|hsa04024\|hsa04510\|hsa05323\|hsa04659\|hsa04380\|hsa04530\|hsa04932\|hsa05161\|hsa04658\|hsa04310\|hsa04915\|hsa05140\|hsa05224\|hsa04921\|hsa05321\|hsa04210\|hsa05418\|hsa04926\|hsa04668\|hsa01522\|hsa04620\|hsa04722\|hsa05142\|hsa04625\|hsa04660\|hsa04933\|hsa04657\|hsa05231\|hsa05132\|hsa04912\|hsa05133\|hsa04012\|hsa05210\|hsa04662\|hsa05120\|hsa04137\|hsa05031\|hsa05211\|hsa05030 | 1:58780788-58784327 | 5.418554 | 2.24E-19 | 2.82E-16 |
| ENSG00000275405 | RF00003 | - | KI270713.1:21861-22024 | 5.36272 | 1.22E-10 | 2.03E-08 |
| ENSG00000090104 | RGS1 | - | 1:192575727-192580031 | 4.942141 | 7.19E-13 | 2.10E-10 |
| ENSG00000214407 | LINC02085 | - | 3:101940859-101997926 | 4.905847 | 3.29E-10 | 4.94E-08 |
| ENSG00000206760 | SNORA6 | - | 3:39408389-39408539 | 4.801447 | 0.001093 | 0.019744 |
| ENSG00000158050 | DUSP2 | hsa04010 | 2:96143166-96145440 | 4.571603 | 7.35E-14 | 2.65E-11 |
| ENSG00000202444 | RNU5E-6P | - | 1:44819883-44819997 | 4.566221 | 0.000146 | 0.004078 |
| ENSG00000254806 | SYS1-DBNDD2 | - | 20:45363200-45410610 | 4.557864 | 3.19E-05 | 0.001189 |
| ENSG00000137801 | THBS1 | hsa05165\|hsa04151\|hsa05206\|hsa04145\|hsa05205\|hsa04510\|hsa04015\|hsa04350\|hsa04512\|hsa04115\|hsa05144\|hsa05219 | 15:39581079-39599466 | 4.52219 | 1.29E-16 | 8.50E-14 |
| ENSG00000163736 | PPBP | hsa04060\|hsa04062 | 4:73987038-73988197 | 4.492555 | 1.12E-09 | 1.47E-07 |
| ENSG00000232810 | TNF | hsa04612\|hsa04650\|hsa05165\|hsa05166\|hsa05169\|hsa05168\|hsa04060\|hsa04010\|hsa05332\|hsa05163\|hsa05164\|hsa05170\|hsa05152\|hsa05322\|hsa04621\|hsa05145\|hsa05205\|hsa05323\|hsa05010\|hsa04380\|hsa04640\|hsa04932\|hsa05161\|hsa04150\|hsa04217\|hsa04940\|hsa05160\|hsa05140\|hsa05330\|hsa05321\|hsa04210\|hsa05418\|hsa04071\|hsa04668\|hsa04064\|hsa04620\|hsa04931\|hsa05142\|hsa05310\|hsa04625\|hsa04660\|hsa04933\|hsa04657\|hsa05146\|hsa05414\|hsa04350\|hsa05133\|hsa05410\|hsa04920\|hsa04622\|hsa04664\|hsa05134\|hsa05014\|hsa04930\|hsa05144\|hsa05143\|hsa01523 | 6:31575567-31578336 | 4.439507 | 3.42E-13 | 1.07E-10 |
| ENSG00000210195 | MT-TT | - | MT:15888-15953 | 4.422931 | 1.62E-12 | 4.12E-10 |
| ENSG00000266698 | MIR3945 | - | 4:184851013-184851110 | 4.348575 | 2.95E-10 | 4.52E-08 |
| ENSG00000230291 | AC078817.1 | - | 12:80102899-80103333 | 4.311645 | 2.84E-12 | 6.83E-10 |
| ENSG00000125740 | FOSB | hsa04380\|hsa05034\|hsa04657\|hsa05031\|hsa05030 | 19:45467995-45475179 | 4.210276 | 8.67E-12 | 1.81E-09 |
| ENSG00000112149 | CD83 | - | 6:14117256-14136918 | 4.16708 | 6.20E-19 | 7.52E-16 |
| ENSG00000226221 | RPL26P19 | - | 5:56504635-56505072 | 4.12138 | 1.04E-12 | 2.85E-10 |
| ENSG00000274210 | RF00003 | - | 1:148522601-148522765 | 4.088702 | 5.76E-13 | 1.71E-10 |
| ENSG00000123358 | NR4A1 | hsa04151\|hsa04010\|hsa04934\|hsa04925\|hsa04927 | 12:52022832-52059507 | 4.087102 | 2.32E-10 | 3.66E-08 |
| ENSG00000176020 | AMIGO3 | - | 3:49716834-49719695 | 4.061277 | 1.28E-06 | 7.88E-05 |
| ENSG00000205038 | PKHD1L1 | - | 8:109362477-109530330 | 4.040333 | 2.59E-09 | 3.16E-07 |
| ENSG00000126945 | HNRNPH2 | - | X:101408295-101414133 | 4.029192 | 1.40E-15 | 7.07E-13 |
| ENSG00000280987 | MATR3 | - | 5:139273752-139331671 | 4.020035 | 9.25E-05 | 0.002778 |
| ENSG00000109475 | RPL34 | hsa03010 | 4:108620566-108630412 | 4.001137 | 8.84E-16 | 4.87E-13 |
| ENSG00000233614 | DDX11L10 | - | 16:11555-14090 | 3.96729 | 8.79E-07 | 5.67E-05 |
| ENSG00000236058 | RPL17P36 | - | 10:118872054-118872602 | 3.955212 | 6.20E-08 | 5.67E-06 |
| ENSG00000113070 | HBEGF | hsa05205\|hsa04915\|hsa01522\|hsa04928\|hsa04912\|hsa04012\|hsa05120\|hsa05219 | 5:140332843-140346631 | 3.951523 | 3.10E-09 | 3.73E-07 |
| ENSG00000221949 | LINC01465 | - | 12:62601751-62603690 | 3.894396 | 0.000179 | 0.004806 |
| ENSG00000173210 | ABLIM3 | hsa04360 | 5:149141483-149260542 | 3.874676 | 6.83E-06 | 0.000337 |
| ENSG00000280143 | AP000892.3 | - | 11:117204967-117210292 | 3.867195 | 5.90E-06 | 0.000298 |
| ENSG00000125538 | IL1B | hsa05168\|hsa04060\|hsa04010\|hsa05332\|hsa05163\|hsa05164\|hsa05152\|hsa04621\|hsa05323\|hsa05010\|hsa04659\|hsa04380\|hsa04640\|hsa04932\|hsa04217\|hsa04940\|hsa05140\|hsa05162\|hsa05321\|hsa05418\|hsa04668\|hsa04064\|hsa04620\|hsa05142\|hsa04625\|hsa04933\|hsa04657\|hsa05146\|hsa05132\|hsa04750\|hsa05133\|hsa05134\|hsa04623\|hsa05144\|hsa05143\|hsa01523\|hsa05020 | 2:112829751-112836903 | 3.865609 | 2.45E-18 | 2.66E-15 |
| ENSG00000244398 | AC116533.1 | - | 11:16974693-16975013 | 3.863798 | 2.55E-11 | 4.86E-09 |
| ENSG00000100906 | NFKBIA | hsa05200\|hsa05166\|hsa05169\|hsa05168\|hsa05163\|hsa05164\|hsa05170\|hsa05203\|hsa05167\|hsa04621\|hsa04024\|hsa04062\|hsa05145\|hsa04659\|hsa04380\|hsa05161\|hsa04658\|hsa05160\|hsa05140\|hsa05162\|hsa04210\|hsa04926\|hsa04668\|hsa04064\|hsa04620\|hsa04722\|hsa04931\|hsa05142\|hsa04625\|hsa04660\|hsa04657\|hsa05215\|hsa05222\|hsa04920\|hsa04622\|hsa05220\|hsa04662\|hsa05120\|hsa05134\|hsa04623\|hsa05131 | 14:35401511-35404749 | 3.826831 | 9.19E-17 | 6.33E-14 |
| ENSG00000238057 | ZEB2-AS1 | - | 2:144517978-144521477 | 3.818177 | 1.82E-11 | 3.60E-09 |
| ENSG00000005961 | ITGA2B | hsa05200\|hsa05165\|hsa04151\|hsa04810\|hsa04510\|hsa04015\|hsa04640\|hsa05418\|hsa04611\|hsa05222\|hsa05414\|hsa04512\|hsa05410\|hsa05412 | 17:44372180-44389505 | 3.817996 | 1.24E-05 | 0.000549 |

Supplementary Table 7. The top 50 down-regulated mRNAs in asthma patients

| gene id | gene name | kegg_id | locus | \|log2FC\| | Pvalue | Qvalue |
| --- | --- | --- | --- | --- | --- | --- |
| ENSG00000167768 | KRT1 | - | 12:52674736-52680407 | 7.06266 | 1.58E-12 | 4.07E-10 |
| ENSG00000215695 | RSC1A1 | - | 1:15659869-15661722 | 6.70788 | 2.59E-06 | 0.000145 |
| ENSG00000196549 | MME | hsa05010\|hsa04640\|hsa04974\|hsa04614 | 3:155024124-155183729 | 6.6822 | 3.73E-30 | 5.66E-26 |
| ENSG00000257878 | AC007298.2 | - | 12:95996521-96011489 | 6.61886 | 1.26E-25 | 3.83E-22 |
| ENSG00000244734 | HBB | hsa05144\|hsa05143 | 11:5225464-5229395 | 6.6075 | 9.73E-16 | 5.27E-13 |
| ENSG00000106714 | CNTNAP3 | - | 9:39072767-39288315 | 6.58579 | 7.97E-27 | 3.02E-23 |
| ENSG00000162747 | FCGR3B | hsa04650\|hsa04145\|hsa05152\|hsa05322\|hsa05150\|hsa04380\|hsa05140 | 1:161623196-161631963 | 6.46215 | 8.10E-21 | 1.23E-17 |
| ENSG00000173868 | PHOSPHO1 | hsa01100\|hsa00564 | 17:49223362-49230766 | 6.32337 | 2.45E-18 | 2.66E-15 |
| ENSG00000157551 | KCNJ15 | hsa04971 | 21:38155549-38307357 | 6.26583 | 3.09E-16 | 1.87E-13 |
| ENSG00000257743 | MGAM2 | hsa01100\|hsa04973\|hsa00500\|hsa00052 | 7:142111749-142222324 | 6.09396 | 9.13E-22 | 1.63E-18 |
| ENSG00000110693 | SOX6 | - | 11:15966449-16739591 | 5.94004 | 3.96E-14 | 1.52E-11 |
| ENSG00000268170 | AC073342.2 | - | 7:143220468-143222267 | 5.91137 | 3.31E-17 | 2.71E-14 |
| ENSG00000163464 | CXCR1 | hsa04060\|hsa04144\|hsa04062\|hsa04072\|hsa05120 | 2:218162845-218166995 | 5.72985 | 2.49E-24 | 6.86E-21 |
| ENSG00000173535 | TNFRSF10C | hsa04060 | 8:23102590-23117437 | 5.65759 | 1.20E-28 | 6.74E-25 |
| ENSG00000255801 | AC092746.1 | - | 12:8548361-8567613 | 5.63823 | 1.12E-15 | 5.98E-13 |
| ENSG00000146122 | DAAM2 | hsa04310 | 6:39792298-39904877 | 5.49064 | 7.91E-11 | 1.36E-08 |
| ENSG00000273333 | AL662884.1 | - | 6:32184733-32185882 | 5.48291 | 0.000153 | 0.004232 |
| ENSG00000158578 | ALAS2 | hsa01100\|hsa00860\|hsa00260 | X:55009055-55031064 | 5.47079 | 2.85E-11 | 5.37E-09 |
| ENSG00000271327 | AC010201.2 | - | 12:89367807-89369301 | 5.38042 | 1.68E-14 | 7.07E-12 |
| ENSG00000183762 | KREMEN1 | - | 22:29073078-29168333 | 5.33236 | 2.16E-17 | 1.87E-14 |
| ENSG00000180871 | CXCR2 | hsa04060\|hsa05163\|hsa04144\|hsa04062\|hsa04072\|hsa05120 | 2:218125289-218137253 | 5.31927 | 1.16E-12 | 3.14E-10 |
| ENSG00000004939 | SLC4A1 | hsa04966 | 17:44248385-44268141 | 5.25996 | 8.25E-11 | 1.40E-08 |
| ENSG00000186529 | CYP4F3 | hsa01100\|hsa00590 | 19:15640897-15662825 | 5.21626 | 9.17E-13 | 2.55E-10 |
| ENSG00000161905 | ALOX15 | hsa01100\|hsa04217\|hsa04726\|hsa00590\|hsa04216\|hsa00591 | 17:4630902-4642294 | 5.15285 | 1.91E-11 | 3.74E-09 |
| ENSG00000154928 | EPHB1 | hsa04360 | 3:134597801-135260467 | 5.07235 | 8.18E-20 | 1.08E-16 |
| ENSG00000213934 | HBG1 | - | 11:5248079-5249859 | 5.06568 | 7.92E-06 | 0.000383 |
| ENSG00000111058 | ACSS3 | hsa01100\|hsa00640 | 12:80936414-81261205 | 5.04415 | 1.28E-14 | 5.81E-12 |
| ENSG00000260078 | AC007342.3 | hsa03008 | 16:53364982-53373083 | 5.02703 | 5.02E-24 | 1.27E-20 |
| ENSG00000170956 | CEACAM3 | - | 19:41796437-41811553 | 4.99788 | 4.63E-29 | 4.67E-25 |
| ENSG00000260495 | AC009148.1 | - | 16:81310731-81313423 | 4.97322 | 7.21E-12 | 1.55E-09 |
| ENSG00000136999 | NOV | - | 8:119416306-119424353 | 4.96458 | 4.50E-12 | 1.03E-09 |
| ENSG00000008516 | MMP25 | hsa04928 | 16:3046681-3060726 | 4.91483 | 2.43E-11 | 4.67E-09 |
| ENSG00000131471 | AOC3 | hsa01100\|hsa00260\|hsa00350\|hsa00410\|hsa00360 | 17:42851184-42858130 | 4.88536 | 9.00E-17 | 6.33E-14 |
| ENSG00000133048 | CHI3L1 | - | 1:203178931-203186749 | 4.7756 | 4.08E-08 | 3.88E-06 |
| ENSG00000261471 | AC092145.1 | - | 16:84594393-84596826 | 4.7664 | 7.41E-18 | 7.02E-15 |
| ENSG00000235105 | AL356968.2 | - | 1:48435967-48437223 | 4.75391 | 6.44E-10 | 9.08E-08 |
| ENSG00000111261 | MANSC1 | - | 12:12326056-12350541 | 4.74177 | 8.03E-16 | 4.50E-13 |
| ENSG00000108244 | KRT23 | hsa04915 | 17:40922696-40937634 | 4.70703 | 4.98E-11 | 8.88E-09 |
| ENSG00000248323 | LUCAT1 | - | 5:91054834-91314547 | 4.68822 | 9.87E-22 | 1.66E-18 |
| ENSG00000264204 | AGAP7P | - | 10:46109621-46131358 | 4.67355 | 3.21E-12 | 7.60E-10 |
| ENSG00000261229 | AC021483.2 | - | 15:79843547-79844304 | 4.66656 | 1.39E-11 | 2.81E-09 |
| ENSG00000253981 | ALG1L13P | - | 8:8236003-8244667 | 4.6599 | 2.28E-13 | 7.51E-11 |
| ENSG00000268119 | AC010615.2 | - | 19:21444241-21463908 | 4.5968 | 1.91E-05 | 0.000781 |
| ENSG00000127954 | STEAP4 | - | 7:88270892-88306891 | 4.5931 | 9.54E-23 | 1.81E-19 |
| ENSG00000284948 | AC107959.4 | - | 8:23084355-23103558 | 4.57483 | 2.36E-15 | 1.16E-12 |
| ENSG00000269001 | AC092070.2 | - | 19:53197111-53214522 | 4.53199 | 2.09E-06 | 0.000119 |
| ENSG00000147454 | SLC25A37 | - | 8:23528805-23575463 | 4.48745 | 5.51E-28 | 2.39E-24 |
| ENSG00000280367 | AP002364.1 | - | 11:90223153-90226538 | 4.48452 | 4.15E-07 | 3.04E-05 |
| ENSG00000115590 | IL1R2 | hsa05166\|hsa04060\|hsa05202\|hsa04640\|hsa05418\|hsa05146\|hsa05215 | 2:101991844-102028544 | 4.47348 | 1.70E-13 | 5.67E-11 |
| ENSG00000281106 | TMEM272 | - | 13:51813347-51845175 | 4.42086 | 1.21E-05 | 0.000539 |

Supplementary Table 8.Top 30 of GO Enrichment

| GO_ID | GO_term | TYPE | diff_gene_in_this_GO | UP_GENE | DOWN_GENE | all_diff_gene_in_all_GO | all_gene_in_this_GO | all_gene_in_all_GO | rich_factor | Pvalue | Qvalue |
| --- | --- | --- | --- | --- | --- | --- | --- | --- | --- | --- | --- |
| GO:0070427 | nucleotide-binding oligomerization domain containing 1 signaling pathway | biological_process | 4 | 3 | 1 | 1249 | 4 | 22360 | 17.90232186 | 8.72E-05 | 0.002522069 |
| GO:0031720 | haptoglobin binding | molecular_function | 8 | 0 | 8 | 1249 | 10 | 22360 | 14.32185749 | 3.69E-07 | 3.05E-05 |
| GO:0035976 | transcription factor AP-1 complex | cellular_component | 4 | 4 | 0 | 1249 | 5 | 22360 | 14.32185749 | 0.000166807 | 0.004344999 |
| GO:1990948 | ubiquitin ligase inhibitor activity | molecular_function | 4 | 4 | 0 | 1249 | 5 | 22360 | 14.32185749 | 0.000166807 | 0.004344999 |
| GO:0070180 | large ribosomal subunit rRNA binding | molecular_function | 4 | 4 | 0 | 1249 | 5 | 22360 | 14.32185749 | 0.000166807 | 0.004344999 |
| GO:0005833 | hemoglobin complex | cellular_component | 9 | 0 | 9 | 1249 | 12 | 22360 | 13.42674139 | 1.23E-07 | 1.11E-05 |
| GO:0031838 | haptoglobin-hemoglobin complex | cellular_component | 8 | 0 | 8 | 1249 | 11 | 22360 | 13.01987044 | 6.39E-07 | 4.86E-05 |
| GO:0055105 | ubiquitin-protein transferase inhibitor activity | molecular_function | 4 | 4 | 0 | 1249 | 6 | 22360 | 11.93488124 | 0.000292421 | 0.006745911 |
| GO:0015671 | oxygen transport | biological_process | 9 | 0 | 9 | 1249 | 15 | 22360 | 10.74139311 | 5.37E-07 | 4.20E-05 |
| GO:0005344 | oxygen carrier activity | molecular_function | 8 | 0 | 8 | 1249 | 14 | 22360 | 10.2298982 | 2.69E-06 | 0.000155332 |
| GO:0022625 | cytosolic large ribosomal subunit | cellular_component | 34 | 34 | 0 | 1249 | 62 | 22360 | 9.817402309 | 4.72E-20 | 3.15E-17 |
| GO:0045741 | positive regulation of epidermal growth factor-activated receptor activity | biological_process | 5 | 3 | 2 | 1249 | 10 | 22360 | 8.951160929 | 0.00022124 | 0.00549867 |
| GO:0000353 | formation of quadruple SL/U4/U5/U6 snRNP | biological_process | 5 | 5 | 0 | 1249 | 10 | 22360 | 8.951160929 | 0.00022124 | 0.00549867 |
| GO:1901724 | positive regulation of cell proliferation involved in kidney development | biological_process | 4 | 1 | 3 | 1249 | 8 | 22360 | 8.951160929 | 0.000744963 | 0.014296039 |
| GO:0022626 | cytosolic ribosome | cellular_component | 61 | 59 | 2 | 1249 | 128 | 22360 | 8.53157526 | 1.23E-31 | 1.07E-27 |
| GO:0006614 | SRP-dependent cotranslational protein targeting to membrane | biological_process | 56 | 56 | 0 | 1249 | 120 | 22360 | 8.354416867 | 7.44E-29 | 3.23E-25 |
| GO:0016176 | superoxide-generating NADPH oxidase activator activity | molecular_function | 5 | 0 | 5 | 1249 | 11 | 22360 | 8.137419026 | 0.000326571 | 0.007434857 |
| GO:0006613 | cotranslational protein targeting to membrane | biological_process | 56 | 56 | 0 | 1249 | 124 | 22360 | 8.084919549 | 2.77E-28 | 8.01E-25 |
| GO:0042788 | polysomal ribosome | cellular_component | 14 | 14 | 0 | 1249 | 31 | 22360 | 8.084919549 | 1.51E-08 | 1.85E-06 |
| GO:0022627 | cytosolic small ribosomal subunit | cellular_component | 27 | 25 | 2 | 1249 | 60 | 22360 | 8.056044836 | 1.15E-14 | 6.21E-12 |
| GO:1904667 | negative regulation of ubiquitin protein ligase activity | biological_process | 4 | 4 | 0 | 1249 | 9 | 22360 | 7.956587492 | 0.001108299 | 0.019619151 |
| GO:0060087 | relaxation of vascular smooth muscle | biological_process | 4 | 2 | 2 | 1249 | 9 | 22360 | 7.956587492 | 0.001108299 | 0.019619151 |
| GO:0045047 | protein targeting to ER | biological_process | 57 | 57 | 0 | 1249 | 134 | 22360 | 7.61516676 | 1.09E-27 | 1.89E-24 |
| GO:0030627 | pre-mRNA 5'-splice site binding | molecular_function | 10 | 10 | 0 | 1249 | 24 | 22360 | 7.459300774 | 2.31E-06 | 0.000143212 |
| GO:0035325 | Toll-like receptor binding | molecular_function | 5 | 2 | 3 | 1249 | 12 | 22360 | 7.459300774 | 0.000467897 | 0.009947392 |
| GO:0072599 | establishment of protein localization to endoplasmic reticulum | biological_process | 57 | 57 | 0 | 1249 | 138 | 22360 | 7.394437289 | 3.64E-27 | 5.26E-24 |
| GO:0000184 | nuclear-transcribed mRNA catabolic process, nonsense-mediated decay | biological_process | 59 | 57 | 2 | 1249 | 145 | 22360 | 7.284393032 | 9.07E-28 | 1.97E-24 |
| GO:0006123 | mitochondrial electron transport, cytochrome c to oxygen | biological_process | 8 | 8 | 0 | 1249 | 20 | 22360 | 7.160928743 | 2.46E-05 | 0.00094957 |
| GO:1990535 | neuron projection maintenance | biological_process | 4 | 4 | 0 | 1249 | 10 | 22360 | 7.160928743 | 0.001590058 | 0.026574502 |
| GO:0006930 | substrate-dependent cell migration, cell extension | biological_process | 4 | 3 | 1 | 1249 | 10 | 22360 | 7.160928743 | 0.001590058 | 0.026574502 |

**Supplementary Table 9.Top 30 of KEGG Enrichment**

| pathway_ID | PATHWAY_DES | diff_gene_in_this_pathway | UP_GENES | DOWN_GENES | diff_gene_in_all_pathway | all_gene_in_this_pathway | all_gene_in_all_pathway | rich_factor | Pvalue | Qvalue |
| --- | --- | --- | --- | --- | --- | --- | --- | --- | --- | --- |
| hsa03010 | Ribosome | 60 | 60 | 0 | 601 | 178 | 8877 | 4.978781 | 1.76E-22 | 5.04E-20 |
| hsa05144 | Malaria | 18 | 8 | 10 | 601 | 56 | 8877 | 4.747623 | 5.57E-08 | 7.96E-06 |
| hsa00910 | Nitrogen metabolism | 5 | 1 | 4 | 601 | 17 | 8877 | 4.34423 | 0.002494 | 0.027432 |
| hsa05219 | Bladder cancer | 12 | 5 | 7 | 601 | 43 | 8877 | 4.121967 | 2.60E-05 | 0.001242 |
| hsa05143 | African trypanosomiasis | 9 | 2 | 7 | 601 | 44 | 8877 | 3.021215 | 0.002198 | 0.027333 |
| hsa04964 | Proximal tubule bicarbonate reclamation | 5 | 4 | 1 | 601 | 25 | 8877 | 2.954077 | 0.016 | 0.097363 |
| hsa04960 | Aldosterone-regulated sodium reabsorption | 7 | 7 | 0 | 601 | 37 | 8877 | 2.794397 | 0.008867 | 0.060383 |
| hsa04668 | TNF signaling pathway | 24 | 17 | 7 | 601 | 130 | 8877 | 2.72684 | 1.51E-05 | 0.001079 |
| hsa05132 | Salmonella infection | 19 | 16 | 3 | 601 | 103 | 8877 | 2.724634 | 9.85E-05 | 0.003129 |
| hsa05142 | Chagas disease (American trypanosomiasis) | 22 | 14 | 8 | 601 | 121 | 8877 | 2.685524 | 3.99E-05 | 0.001428 |
| hsa05012 | Parkinson disease | 28 | 23 | 5 | 601 | 161 | 8877 | 2.568762 | 1.06E-05 | 0.001006 |
| hsa04260 | Cardiac muscle contraction | 14 | 12 | 2 | 601 | 82 | 8877 | 2.521773 | 0.001431 | 0.022733 |
| hsa04210 | Apoptosis | 26 | 16 | 10 | 601 | 153 | 8877 | 2.51 | 3.03E-05 | 0.001237 |
| hsa05131 | Shigellosis | 11 | 9 | 2 | 601 | 66 | 8877 | 2.46173 | 0.004808 | 0.041669 |
| hsa05418 | Fluid shear stress and atherosclerosis | 24 | 16 | 8 | 601 | 150 | 8877 | 2.363261 | 0.000146 | 0.004161 |
| hsa05210 | Colorectal cancer | 14 | 11 | 3 | 601 | 88 | 8877 | 2.349834 | 0.002833 | 0.028938 |
| hsa04620 | Toll-like receptor signaling pathway | 20 | 13 | 7 | 601 | 128 | 8877 | 2.307872 | 0.000623 | 0.012736 |
| hsa04971 | Gastric acid secretion | 12 | 8 | 4 | 601 | 77 | 8877 | 2.301878 | 0.006092 | 0.049782 |
| hsa05134 | Legionellosis | 12 | 8 | 4 | 601 | 77 | 8877 | 2.301878 | 0.006092 | 0.049782 |
| hsa04923 | Regulation of lipolysis in adipocytes | 9 | 7 | 2 | 601 | 58 | 8877 | 2.291956 | 0.014976 | 0.095183 |
| hsa04657 | IL-17 signaling pathway | 17 | 16 | 1 | 601 | 111 | 8877 | 2.262131 | 0.001793 | 0.024414 |
| hsa04216 | Ferroptosis | 6 | 4 | 2 | 601 | 40 | 8877 | 2.215557 | 0.042603 | 0.196525 |
| hsa00770 | Pantothenate and CoA biosynthesis | 3 | 0 | 3 | 601 | 20 | 8877 | 2.215557 | 0.09824 | 0.330548 |
| hsa05222 | Small cell lung cancer | 15 | 10 | 5 | 601 | 101 | 8877 | 2.193621 | 0.00416 | 0.037181 |
| hsa04012 | ErbB signaling pathway | 13 | 10 | 3 | 601 | 88 | 8877 | 2.181988 | 0.007281 | 0.052061 |
| hsa05213 | Endometrial cancer | 9 | 5 | 4 | 601 | 61 | 8877 | 2.179237 | 0.020728 | 0.118563 |
| hsa04064 | NF-kappa B signaling pathway | 19 | 10 | 9 | 601 | 129 | 8877 | 2.175483 | 0.001669 | 0.02387 |
| hsa05160 | Hepatitis C | 25 | 11 | 14 | 601 | 170 | 8877 | 2.172115 | 0.000399 | 0.008776 |
| hsa04932 | Non-alcoholic fatty liver disease (NAFLD) | 26 | 21 | 5 | 601 | 178 | 8877 | 2.157472 | 0.000349 | 0.008315 |
| hsa04380 | Osteoclast differentiation | 28 | 17 | 11 | 601 | 192 | 8877 | 2.154014 | 0.000225 | 0.005841 |


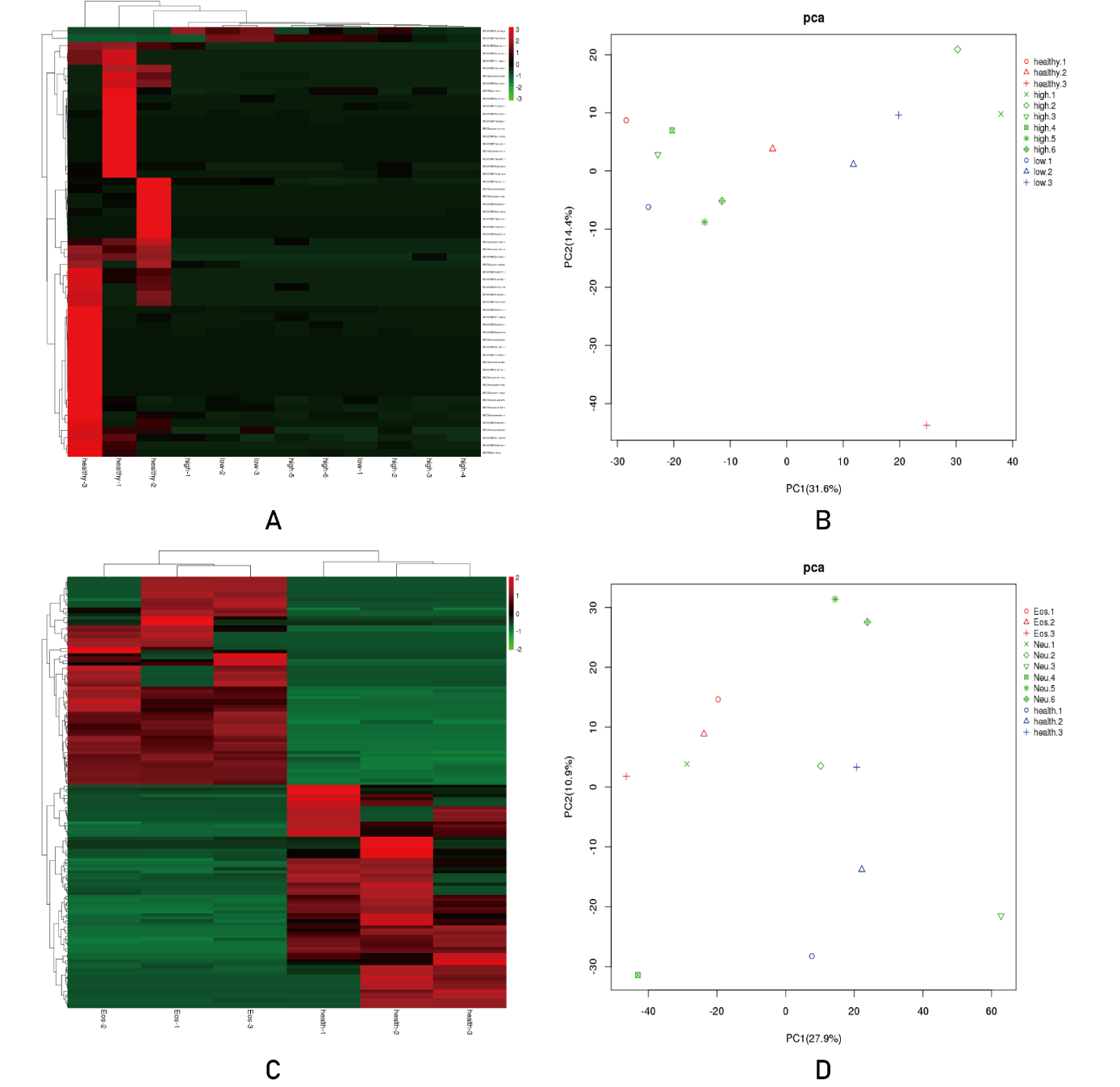


Supplementary Figure 1. Differential expression of lncRNAs and PCA in GSE106230 and GSE117038 databases
